# Evidence of a Demethylase-Independent Role for the H3K4-Specific Histone Demethylases in *Aspergillus nidulans* and *Fusarium graminearum* Secondary Metabolism

**DOI:** 10.3389/fmicb.2019.01759

**Published:** 2019-08-13

**Authors:** Simone Bachleitner, Jens Laurids Sørensen, Agnieszka Gacek-Matthews, Michael Sulyok, Lena Studt, Joseph Strauss

**Affiliations:** ^1^Department of Applied Genetics and Cell Biology, University of Natural Resources and Life Sciences, Vienna (BOKU), Vienna, Austria; ^2^Department of Biotechnology, Chemistry and Environmental Engineering, Aalborg University, Aalborg, Denmark; ^3^Department for Agrobiotechnology (IFA-Tulln), Institute of Bioanalytics and Agro-Metabolomics, University of Natural Resources and Life Sciences, Vienna (BOKU), Vienna, Austria

**Keywords:** KDM5, histone demethylase, secondary metabolism, *Aspergillus nidulans*, *Fusarium graminearum*, H3K4 methylation, CclA, SppA

## Abstract

Fungi produce a plethora of secondary metabolites (SMs) involved in cellular protection, defense, and signaling. Like other metabolic processes, transcription of SM biosynthesis genes is tightly regulated to prevent an unnecessary use of resources. Genes involved in SM biosynthesis are usually physically linked, arranged in secondary metabolite gene clusters (SMGCs). Research over the last decades has shown that chromatin structure and posttranslational modifications (PTMs) of histones represent important layers of SMGC regulation. For instance, trimethylation of histone H3 lysine 4 (H3K4me3) is a PTM typically associated with promoter regions of actively transcribed genes. Previously, we have shown that the H3K4me3-specific, JmjC domain-containing histone demethylase KdmB functions not only in repression but also in activation of secondary metabolism in *Aspergillus nidulans*, suggesting that KdmB has additional functions apart from histone demethylation. In this study, we identified demethylase-independent functions of KdmB in transcriptional regulation of SM gene clusters. Furthermore, we show that this activating and demethylase-independent role of the H3K4 demethylase is also conserved in the phytopathogenic fungus *Fusarium graminearum*. Lack of FgKdm5 resulted in significant downregulation of five of seven analyzed SMs, whereby only one SMGC depends on a functional JmjC-domain. In *A. nidulans* strains deficient in H3K4 methylation, i.e., *cclA*∆, largely phenocopied *kdmB*∆, while this is not the case for most of the SMs analyzed in *Fusarium* spp. Notably, KdmB could not rescue the demethylase function in ∆*fgkdm5* but restored all demethylase-independent phenotypes.

## Introduction

Filamentous fungi produce a plethora of structurally diverse low molecular weight compounds, so-called secondary metabolites (SMs), that may provide a competitive advantage for the producing organism (Macheleidt et al., 2016). Among SMs are potent toxins that frequently occur in contaminated food and feed (Streit et al., 2013), but also pharmaceuticals and plant hormones that are applied in medicine and agriculture, respectively (Dreyfuss et al., 1976; Anke et al., 1977; Rademacher, 1997; Manzoni and Rollini, 2002). SMs are synthesized by stepwise enzymatic reactions. Enzymes involved in the biosynthesis are usually encoded in physically linked SM gene clusters (SMGCs). To prevent an unnecessary use of resources, the expression of SM genes is tightly regulated. Each SMGC requires defined environmental or developmental conditions to be induced (Macheleidt et al., 2016). One advantage of SM gene clustering could be the possibility to co-regulate them by large-scale chromatin structure that would grant or deny access to underlying regulatory elements (Gacek and Strauss, 2012; Pfannenstiel and Keller, 2019). Chromatin is composed of histones and chromatin-associated factors. Histones are modified by posttranslational chemical modifications (e.g., acetylation, methylation, or phosphorylation) on different residues, and a certain combination of these histone marks generates a recognition platform for chromatin regulators that strongly influence how the underlying genetic information is read (Jenuwein, 2002; Wood et al., 2005; Drelon et al., 2018). The combination of histone PTMs defines the overall chromatin structure and transcriptional activity at a given genomic region. Chromatin-based gene silencing relies on heterochromatic structures that are characterized by densely packed nucleosomes, whereas loosely arranged nucleosomes in euchromatin are open for transcriptional activation. Both chromatin states are associated with the activity of certain histone-modifying enzymes that are often part of large complexes involved in the recognition, addition, or removal of histone PTMs (Kouzarides, 2007; Piunti and Shilatifard, 2016). Notably, many histone PTMs recruit additional chromatin-modifying enzymes that further remodel the chromatin landscape, thereby enabling cells to respond dynamically to environmental or developmental cues (Gacek and Strauss, 2012; Pfannenstiel and Keller, 2019).

One prominent euchromatic histone PTM is trimethylation of lysine 4 on histone H3 (H3K4me3). H3K4 methylation is established by the COMPASS complex (complex of proteins associated with Set1) that is conserved in eukaryotes (Miller et al., 2001; Krogan et al., 2002; Hughes et al., 2004; Lee et al., 2007; Wu et al., 2008; Jiang et al., 2011; Shilatifard, 2012). H3K4 methylation is also associated with active transcription of the majority of genes in *Aspergillus* spp. and *Fusarium* spp. (Connolly et al., 2013; Wiemann et al., 2013; Gacek-Matthews et al., 2016). Notably, while H3K4 methylation is generally associated with transcriptional gene activation, COMPASS-mediated silencing of genes located near telomeres has also been described (Briggs et al., 2001; Krogan et al., 2002; Bok et al., 2009; Mikheyeva et al., 2014). In agreement with these findings, deletion of the COMPASS components *cclA* and *CCL1* (homologous to *bre2*) in *Aspergillus* spp. and *Fusarium* spp., respectively, resulted in increased expression of several SM cluster genes and the subsequent production of additional SMs in these fungi (Bok et al., 2009; Palmer et al., 2013; Shinohara et al., 2016; Studt et al., 2017). Noteworthy, in *Aspergillus nidulans* as well as in *Fusarium graminearum* and *Fusarium fujikuroi*, SMGCs are largely devoid of this histone PTM, even under activated conditions (Connolly et al., 2013; Wiemann et al., 2013; Gacek-Matthews et al., 2016; Studt et al., 2017). H3K4 methylation is countered by enzymes of the KDM5-family of histone demethylases, which are able to remove one or two methyl groups from H3K4me3. Their function has thus mainly been associated with transcriptional repression. However, there is also evidence for transcriptional activation by KDM5-family members, e.g., in mammalian systems and *Drosophila melanogaster* (Klose et al., 2007; Secombe et al., 2007; Secombe and Eisenman, 2007; Seward et al., 2007). Recently, we have shown that the *A. nidulans* KDM5 homolog KdmB balances H3K4 trimethylation levels and both positively and negatively influences around one third of the transcriptome under the tested growth conditions. Notably, around 50% of SM-related genes are affected by *kdmB* deletion, and the vast majority (80%) of them show reduced expression in the *kdmB* deletion mutant (Gacek-Matthews et al., 2016). This bipartite, locus-dependent activating or repressing function seems to be conserved also in *F. fujikuroi* (Janevska et al., 2018). Thus, it is intriguing to hypothesize that the activating functions of KdmB are also conserved also conserved in fungi and that SM-related genes are major targets of this multi-domain chromatin regulator.

KDM5 proteins are composed of multiple domains, which are necessary for their diverse role in transcriptional regulation. In detail, KdmB is a member of the Jumonji C (JmjC) domain-containing demethylases, catalyzing lysine demethylation of histones through an oxidative reaction that requires iron (Fe^2+^) and α-ketoglutarate as substrates (Klose et al., 2006). Additionally to its catalytic JmjC-domain (IPR003347), KdmB harbors a putative JmjN domain (IPR003349), an AT-rich interaction domain (ARID, IPR001606), a C5HC2-type zinc finger motif (IPR004198), and two plant homeo domains (PHD, IPR001606). Recently Liu and Secombe (2015) have shown that the KDM5 homolog Lid in *D. melanogaster* activates gene expression independently of its demethylase domain by recognizing the chromatin context through its PHD domains. In fact, the N-terminal PHD domain is able to read unmethylated lysine residues, whereas the C-terminal PHD domain binds to H3K4me2/me3 marks (Li et al., 2010). Here, we provide evidence for a demethylase-independent role of KdmB in secondary metabolism in *A. nidulans*. Notably, lack of H3K4 methylation, which was engineered by removal of crucial COMPASS components (CclA, SetA) or by replacement of H3K4 by arginine (H3K4R), largely phenocopied the *kdmB* deletion with regard to SMGC regulation and SM production. Furthermore, we show that both the demethylase-dependent and independent role of KDM5-family members in SM gene regulation extends to other fungi like the plant pathogen *F. graminearum.* However, functional differences must exist between both homologs, as loss of the demethylase function in *F. graminearum* ∆*fgkdm5* strains is not rescued by expression of *A. nidulans kdmB*.

## Materials and Methods

### Fungal Strains, Media, and Growth Conditions

The wild-type strains of *A. nidulans* (AnWT) pabaA1 veA1 (Pontecorvo et al., 1953) and *F. graminearum* (FgWT) PH-1 (FGSC 9075, NRRL 31084) were used as parental strains for deletion and targeted mutagenesis experiments. For protoplasting, DNA isolation, and western blot analyses, all strains were grown in darkness for 3 days on solid complete media (CM) (Pontecorvo et al., 1953) covered with cellophane sheets (Folia Bringmann) at 37 and 20°C in case of *A. nidulans* and *F. graminearum*, respectively. Fungal growth test were performed either on solid minimal medium (FMM: *F. graminearum*; AMM: *A. nidulans*; Todd et al., 2007), complete medium (FCM: *F. graminearum;* ACM*: A. nidulans*), or complete medium supplemented with 1 M sorbitol (ACMS: *A. nidulans*; Govindaraghavan et al., 2014). For *A. nidulans* gene expression and SM analyses, the respective strains were grown in liquid Aspergillus minimal media (AMM; Todd et al., 2007) in darkness at 37°C for 48 h. In case of *F. graminearum*, SM analyses were performed with strains grown on potato dextrose agar (PDA) in darkness at 25°C for 2 weeks (Giese et al., 2013). Cultivation of *Saccharomyces cerevisiae* and *Escherichia coli* was performed as previously described (Schumacher, 2012).

### Plasmid Constructions

If not mentioned otherwise, plasmid generations were performed using yeast recombinational cloning as described by Schumacher (2012). All primers used for polymerase chain reaction (PCR) were obtained from Sigma-Aldrich GmbH. For all generated constructs, the strategy and primers used in this study can be found in Supplementary Tables S1, S2. Generally, for deletion constructs, the 5′ and 3′ flank fragments were amplified with the primer pairs 5F//5R and 3F//3R from genomic DNA of the respective parental wild-type strain (AnWT, FgWT). For *A. nidulans* constructs, auxothrophic marker cassettes (*pyrG* or *riboB*) were used as selectable markers and amplified from *A. fumigatus* genomic DNA. For *F. graminearum* the *hph* or *neoR* resistance cassette was amplified from template pCSN44 (Staben et al., 2017) and p*fgccl*-IL (Studt et al., 2017), respectively. Constructs for targeted mutagenesis were generated by using primers harboring the desired mutation. Complementation constructs were generated by amplifying the wild-type gene *kdmB/FgKDM5* from parental genomic DNA. In case of *A. nidulans, kdmB* was driven by its native promotor and terminator sequences, whereas for *F. graminearum,* the *FgKDM5* gene was driven by the native promotor and fused to the glucanase terminator of *Botrytis cinerea* (BcTgluc) from pNAH-P*oliC*::*bcltf3*-*gfp* (Brandhoff et al., 2017). For cross-complementation constructs, the *kdmB* gene was amplified from *A. nidulans* genomic DNA driven by the native *Fusarium* promotor. The *kdmB* gene was fused to BcTgluc, followed by *neoR* amplified from p*fgccl*-IL (Studt et al., 2017). *S. cerevisiae* FY834 was transformed with the obtained fragments and the *Eco*RI/*Xho*I-restricted pRS426 yielding the plasmids pkdmB*, p*kdmB*Δ/*kdmB*^Cis^, p*cclA*Δ, p*sppA*Δ, pΔ*fgkdm5,* pFgKDM5*, pΔ*fgkdm5/FgKDM5*^Ces^, and pΔ*fgkdm5/kdmB*^Cis^. A proof-reading polymerase was used for amplification of all plasmid parts, and correct assembly of the gained plasmids was verified by restriction digest and/or sequencing. All primers used for plasmid generation and strain generation strategies are listed in Supplementary Tables S1, S2.

### Fungal Transformations

In case of *A. nidulans,* experimental strains were obtained by transformation into an *nkuA*Δ strain, which reduces the frequency of non-homologous integration (Nayak et al., 2006). Fungal transformations were performed essentially as described (Tilburn et al., 1983; Studt et al., 2017). In case of *A. nidulans,* the constructs pKdmB* and p*kdmB*Δ/*kdmB*^Cis^ were linearized with *Mlu*I, whereas for p*cclA*Δ and p*sppA*Δ, the knockout fragments were amplified prior transformation with the primers An_CclA_del_1F//An_CclA_del_02R and An_SppA_del_F1//An_SppA_del_R2. In case of *F. graminearum*, we used the split marker approach (Goswami, 2012). For deletion/mutation of *kdm5/FgKDM5* the fragments were amplified from pΔ*fgkdm5/*pΔ*fg*Kdm5* with the primers Kdm5_5F//Split-mark_hphF and Split-mark_hphR//Kdm5_3R. For complementation of Δ*fgkdm5,* the plasmid Δ*fgkdm5/FgKDM5* was integrated ectopically into the Δ*fgkdm5* strain. For cross-complementation, the fragments were amplified from pΔ*fgkdm5/kdmB*^Cis^ with the primers Kdm5_5F//GeniSplitF and GeniSplitR//Kdm5_3R. Transformed protoplasts were regenerated as described by Studt et al. (2017).

In case of *A. nidulans* KdmB* mutants, homologous recombination events were checked by using the primers ribo_fum_check_F//8211_downst_R (Supplementary Figure S6). For further verification of the mutation H642G and E644Q, a fragment was amplified with the primers F9//R8 and sequenced. For *kdmB*Δ*/kdmB*^Cis^ mutants, homologous recombination events were verified by using the primers dia_kdmB_F02//kdmB_comp_R02 and dia_pyrG_R01//dia_kdmB_F01 (Supplementary Figure S6). The knock-out mutants *cclA*Δ and *sppA*Δ were checked using the primers dia_dCclA_UP_F//dia_pyrG_fum_5 and dia_SppA_UP_F//dia_pyrG_fum_5′ for the upstream region and dia_dCclA_DownR//dia_pyrG_fum_3′ and dia_dSppA_DownR//dia_pyrG_fum_3′ for the downstream region. The absence of the wild-type genes, *cclA* and *sppA*, was checked using the primer pairs dia_WT_CclA_F//dia-WT_CclA_R and dia_WTSppA_F//dia_WTSppA_R, respectively (Supplementary Figure S2).

In case of *F. graminearum,* homologous recombination events of the Δ*fgkdm5* mutants were verified by the primer pairs dia_kdm5_F//pCSN44_hph-trpcT as well as dia_kdm5_R//pCSN44_trpC_P2 (Supplementary Figure S8). FgKdm5* mutants were checked with the primer pairs dia_Kdm5_F//Kdm5_DM_UP_R for the upstream fragment and dia_Kdm5_R//pkS_GenR_F for the downstream fragment (Supplementary Figure S8). Δ*fgkdm5/kdmB*^Cis^ mutants were checked with the primer pairs dia_Kdm5_F//KdmB_dia_check_UP_R and dia_Kdm5_R//pks_genR_F (Supplementary Figure S10). Δ*fgkdm5/FgKDM5*^Ces^ mutants were verified with the primer pair Kdm5_HK_F/Kdm5_HK_R (Supplementary Figure S8).

### Standard Molecular Techniques

For DNA isolation, lyophilized mycelium was grounded to a fine powder, re-suspended in extraction buffer, and isolated as previously described (Tilburn et al., 1983; Cenis, 1992). Isolated genomic DNA was used for PCR amplification and Southern blot analysis. Deletion, complementation, and cross-complementation fragments were amplified with the proof-reading Phusion High-Fidelity DNA Polymerase (Thermo Fisher Scientific), and PCR reactions were set up accordingly to the manufacturers’ protocol. For diagnostic PCRs, the GoTaq® Green Master Mix (Promega) was used, and the PCR reactions were set up according to the users’ manual. For Southern, genomic DNA of *cclA*Δ, *sppA*Δ and Δ*fgkdm5* was digested with the enzymes *Xho*I, *Eco*32I and *Hin*dIII, respectively. The digested DNA was separated on a 1% (w/v) agarose gel and transferred onto positively charged nylon membranes (Roche Diagnostics GmbH, Germany) by downward blotting. Probes were labeled with DIG-11-dUTP using the DIG-High Prime DNA Labeling and Detection Starter Kit II from Roche. Primers used for probe generation are listed in Supplementary Table S1.

For expression analyses, RNA was extracted from lyophilized mycelium using the TRIzol Reagent (Thermo Fisher Scientific) according to the manufacturers’ instruction. For cDNA synthesis, 1 μg of total RNA was treated with DNaseI (Thermo Fisher Scientific) and subsequently reversely transcribed using the iScript™ cDNA Synthesis Kit (BioRad). Generated plasmids were extracted and purified from *E. coli* and *S. cerevisiae* with the GeneJET™ plasmid miniprep kit (Fermentas GmbH, St. Leon-Rot, Germany). Sequencing of each plasmid was performed using primers listed in Supplementary Table S1. For western blot analyses, mycelium from 2 to 3 days old strains was ground to a fine powder, and proteins were extracted as described (Studt et al., 2016). Roughly, 10 μg (*A. nidulans*) and 15 μg (*F. graminearum*) of proteins were used for SDS-Page and subsequent western blotting. The membrane was probed with 1:4,000 dilutions of H3 C-Term (Active Motif), H3K4me1 (Active Motif, AM61633), H3K4me2 (Active Motif, AM 39141), and H3K4me3 (Active Motif, AM 39159 for *F. graminearum*, Abcam, ab8580 for *A. nidulans*) primary antibodies and 1:10,000 dilutions of anti-rabbit (Sigma A0545) HRP conjugated secondary antibody. Chemoluminescence was detected with Clarity™ ECL Western Substrate and ChemDoc™ XRS (Bio-Rad). Densitometric quantification of western blot signals was performed with the ImageJ software and normalized to the histone H3 C-term signal. Subsequently, the signal of the according wild type was set to a value 1; consequently, the presented results are the fold change to the control reaction. Each western blot was performed at least three times for each set of samples.

### Chemical Analyses

*A. nidulans* strains were grown for 48 h in liquid AMM under SM inducing conditions (1% glucose, 10 mM NaNO_3_), and the supernatant was analyzed. The samples were run on a QTrap 5500 LC–MS/MS System (Applied Biosystems, Foster City, CA, USA) equipped with a TurboIonSpray electrospray ionization (ESI) source and a 1290 Series HPLC System (Agilent, Waldbronn, Germany). Chromatographic separation was done at 25°C using a Gemini C18 150 × 4.6 mm i.d., 5 μm particle size, equipped with a C18 3 × 4 mm i.d. security guard cartridge (Phenomenex, Torrance, CA, USA). The chromatographic method and chromatographic and mass spectrometric parameters are described elsewhere (Malachová et al., 2014).

*F. graminearum* strains were grown for 2 weeks on PDA plates and subsequently extracted using MeOH/CH_2_Cl_2_/EtOAc (1/2/3, v/v), evaporated, and resuspended in methanol/H_2_O (1/1, v/v) as previously described (Sondergaard et al., 2016). Extracts from the FgWT and two independent *fgkdm5* deletion mutants were initially analyzed by high-resolution mass spectrometry in positive ionization mode as previously described (Wollenberg et al., 2016). Quantitative analyzes of known SMs from *F. graminearum* were performed on a Thermo Vantage triple stage quadrupole mass spectrometer (Thermo Fisher Scientific, San José, CA, USA) with a heated electrospray ionization probe using the chromatography and MS settings for deoxynivalenol, zearalenone, fusarin C, aurofusarin, fusaristatin A, fusarielin H, and chrysogine as previously described (Sørensen and Sondergaard, 2014; Wollenberg et al., 2017).

### Reverse Transcriptase-Quantitative Polymerase Chain Reaction

RT-qPCR was performed with iQ SYBR Green Supermix (Bio-Rad, Munich, Germany) using an iCycler iQ Real-Time PCR System (Bio-Rad). To quantify mRNA levels of SMGCs in *A. nidulans*, the following primers were used: PEN gene cluster (q_ipnA_F//q_ipnA_R), ST gene cluster (q_aflR_F//q_aflR_R), ORS gene cluster (q_orsA_F//q_orsA_R), and the MDP gene cluster (q_mdpG_F//q_mdpG_R). To quantify the expression level of *kdmB,* the primers q_kdmB_F//q_kdmB_R were used. Levels of mRNA were related to constitutively expressed reference genes, i.e., AN6838 encoding β-tubulin (q_TUB_F//q_TUB-R) and AN0290 encoding actin (q_ActA_F//q_ActA_R).

In case of *F. graminearum*, the following primers were used for *FgKDM5* expression (q_Kdm5_F//q_Kdm5_R). Levels of mRNA were related to following reference genes: *FGSG_06257* encoding glyceraldehyde 3-phosphate dehydrogenase (GAPDH) (GAPDH_qPCR_fwd//GAPDH_qPCR_rev), *FGSG_07335* encoding actin (qPCR_actin_F// qPCR_actin_R), and *FGSG_09530* encoding ß-tubulin (cDNA_ß-TUB_F //cDNA_ß-TUB_R). Primer efficiencies in the RT-qPCR were kept between 90 and 110%. Relative expression levels were calculated using the ∆∆Ct method (Pfaffl, 2001). Experiments were performed in biological and technical duplicates. Primer sequences are listed in Supplementary Table S1.

## Results

### Lack of COMPASS Components Largely Phenocopies KdmB With Regard to Secondary Metabolite Gene Regulation

In a previous study, we have shown that KdmB regulates the H3K4 methylation levels and plays a central role in secondary metabolism in *A. nidulans* (Gacek-Matthews et al., 2016). Here, the majority of SMGCs (11 out of 18 differentially expressed SMGCs) are less expressed in a strain deleted for *kdmB* compared to the *A. nidulans* wild-type strain (AnWT) under SM cultivation conditions. However, H3K4me3 is under-represented in chromatin landscapes of SMGCs and, if present, H3K4me3 levels are not increased but decreased at these sites in *kdmB*∆. This suggests that KdmB promotes SM gene expression either by regulating SM genes independently of H3K4me3 or indirectly by targeting genes of *trans*-acting factors. To study the relevance of H3K4 methylation for KdmB-mediated regulation, we have now analyzed mutants altered in H3K4 methylation (i.e., H3K4R, *setA*∆, *cclA*∆, *sppA*∆) for their characteristics in SM biosynthesis. Strains deficient in H3K4 methylation, i.e., a strain in which H3K4 has been replaced by arginine (H3K4R) as well as a strain lacking SetA, the catalytic subunit of the COMPASS complex homologous to the histone methyltransferase Set1 are very sick (Govindaraghavan et al., 2014). Also in our hands both strains featured retarded hyphal growth (20% of AnWT) on solid Aspergillus complete medium (ACM) and did not grow at all on solid (Supplementary Figure S1) or in liquid Aspergillus minimal medium (AMM, data not shown). Thus, these strains could not rationally be included in the present study to analyze the impact of H3K4 methylation on SM gene regulation in *A. nidulans*.

**Figure 1:**
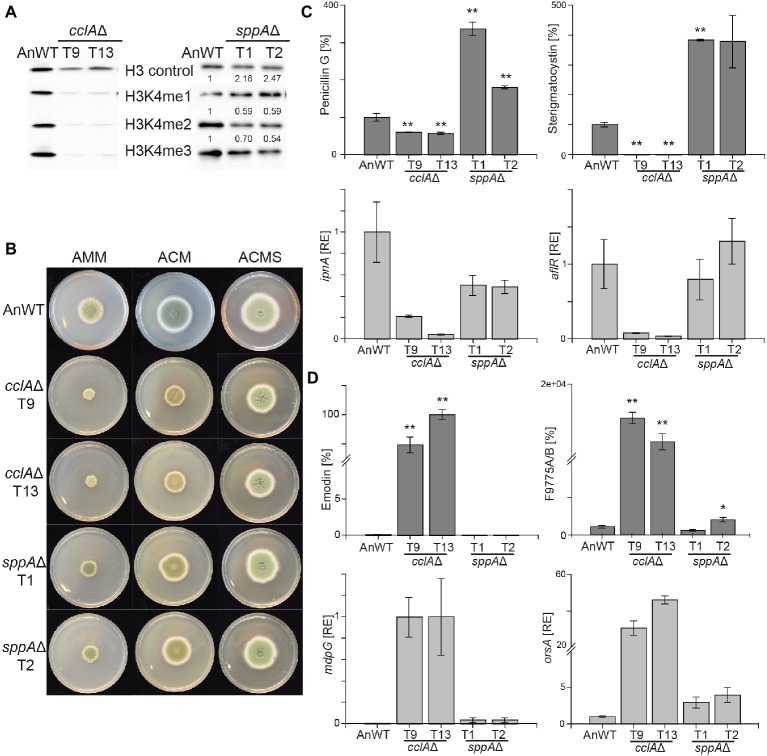
Lack of *cclA*Δ and *sppA*Δ result in decreased global H3K4me3 and altered SM biosynthesis in *Aspergillus nidulans*. **(A)** The *A. nidulans* wild-type strain (AnWT) and the respective mutant strains were grown in liquid AMM cultures with 10 mM sodium nitrate for 48 h prior to protein extraction. Whole protein extracts were subsequently isolated from lyophilized mycelia, and 15 μg of proteins were used for western blot analysis. H3 C-Term, H3K4me1/me2 and me3 antibodies were used for detection. A densitometric analysis was performed, and the respective wild-type strain was arbitrarily set to 1. **(B)** For radial growth analysis, AnWT, *cclA*Δ, and *sppA*Δ strains were grown on solid AMM, ACM, and ACMS plates at 37°C under dark conditions. Pictures were taken after 5 days of growth. Experiments were done in triplicates. **(C-D)** AnWT, *cclA*Δ, and *sppA*Δ were grown in liquid AMM medium with 10 mM sodium nitrate at 37°C and 180 rpm for determination of penicillin G, sterigmatocystin, emodin, and F9775A/B. Supernatants of 48 h old liquid cultures were applied for HPLC-MS/MS measurements. To exclude falsifications in SM biosynthesis due to differences in hyphal growth, SM production was correlated to biomass formation. For a direct comparison, production of AnWT was arbitrarily set to 100%. For statistical analysis, a student’s *t* test was performed: ^*^p < 0.05; ^**^p < 0.01; n.d., not detected. For determination of transcript levels, RNA was extracted from 48 h old, lyophilized mycelia. The transcription levels of *ipnA* [encoding the non-ribosomal peptide synthetase (NRPS) required for penicillin (PEN) production], *aflR* [encoding the pathway-specific transcription factor for sterigmatocystin (ST) biosynthesis], *orsA* [encoding a non-reducing polyketide synthase (NR-PKS) required for orsellinic acid and F9775A/B biosynthesis], and *mdpG* (encoding a NR-PKS required for emodin biosynthesis) were determined *via* RT-qPCR. Experiments were done in triplicates. For comparison, expression levels of AnWT were arbitrarily set as 1. Mean values and standard deviations are shown. RE, relative expression.

Two additional COMPASS components have been described to influence the H3K4 methylation status, namely the subunits Bre2 and Spp1, both required for efficient H3K4 trimethylation in *S. cerevisiae* (Roguev et al., 2001; Dehé et al., 2006; Acquaviva et al., 2013). Based on the phenotype of reverse genetic screens, the *Aspergillus* homologs of Bre2 were designated CclA and found to participate in repression of subtelomeric SMGCs in *A. nidulans* (Bok et al., 2009) and *A. fumigatus* (Palmer et al., 2013). The Spp1 homolog in *Aspergillus*, SppA, has only been characterized so far in *A. oryzae* where it is necessary for full H3K4 trimethylation (Shinohara et al., 2016). We identified the Spp1 homolog in the *A. nidulans* genome database by BlastP analysis as AN2850 (E-value of 2.0e-17). We deleted both *cclA* and *sppA* in the same genetic background and at least two independent mutants were verified by diagnostic PCR and Southern blot analysis (Supplementary Figure S2). Western analysis with antibodies directed against different methylation forms of H3K4 showed that lack of CclA leads to a severe reduction in mono-, di-, and trimethylation of this residue *in vivo,* whereas the absence of SppA reduced H3K4 tri- and dimethylation by roughly 50% with a concomitant increase of H3K4 monomethylation by a factor of two (Figure 1A). Contrary to *set1*Δ and H3K4R, the *cclA*Δ and *sppA*Δ strains did not show such extreme growth phenotypes. Deletion of *sppA* did not detectably alter growth characteristics when compared to AnWT, while *cclA*∆ mutant featured around 50% reduced radial growth on solid complete and minimal media and around 30% reduced biomass formation in liquid AMM shake cultures (Figure 1B; Supplementary Figure S3). To sum up, the strongest phenotype with regard to radial growth was observed for H3K4R followed by *setA*∆ and *cclA*∆, all being significantly impaired in H3K4 methylation, while deletion of *sppA* had only mild impact on H3K4 methylation levels and growth.

To analyze if strains with reduced or abolished H3K4me3 levels affect secondary metabolism in *A. nidulans* in a similar manner as strains lacking the H3K4 demethylase KdmB, the generated mutant strains were grown under SM-inducing conditions (liquid AMM with 10 mM sodium nitrate) and analyzed for SMs known to be regulated by this chromatin mark. Biosynthesis of both penicillin G and sterigmatocystin were almost abolished in strains lacking CclA. This is similar although not identical to what has been found in *kdmB*∆. In agreement with the reduced production of the metabolites, also the expression of the biosynthetic genes, i.e., *ipnA* (encoding the non-ribosomal peptide synthetase (NRPS) required for penicillin (PEN) production) and *aflR* (encoding the pathway-specific transcription factor for sterigmatocystin (ST) biosynthesis) were reduced (Figure 1C). This indicates that H3K4 methylation and KdmB are required for PEN and ST biosynthesis. Contrary to *cclA*∆, the lack of SppA did not reduce the production of the two model SMs. Notably, the levels of penicillin G (200% of AnWT) and sterigmatocystin (300% of AnWT) were even found to be increased in *sppA*∆ compared to AnWT (Figure 1C). However, transcript levels for the tested cluster genes, i.e., *ipnA* (PEN cluster) and *aflR* (ST cluster) were basically unchanged in comparison to AnWT indicating that the altered COMPASS complex without the SppA subunit and the concomitantly slightly reduced overall H3K4 methylation levels are not crucial for PEN and ST cluster transcription (Figure 1C).

We also tested to which extent the slightly or severely altered global H3K4 methylation levels in *cclA*∆ and *sppA*∆ mutant strains affect KdmB-mediated SMGC repression. For this, we analyzed emodin/monodictyphenone and F9775A/B (yellow polyketides), which are basically not produced in AnWT under our standard laboratory growth conditions (Cluster et al., 2010; Nützmann et al., 2011; Klejnstrup et al., 2012). Consistent with our previous findings, transcription of *orsA* (encoding a non-reducing polyketide synthase (NR-PKS) required for orsellinic acid and F9775A/B biosynthesis) and *mdpG* (encoding a NR-PKS required for emodin biosynthesis) were strongly de-repressed in the *cclA*Δ strain resulting also in high levels of F9775A/B and emodin (Figure 1D). The metabolite levels in the *sppA*Δ strain, however, were almost unchanged; only a slight de-repression was evident at the transcriptional as well as at the metabolite level. Taken together, loss of CclA (but not SppA) largely phenocopied *kdmB*∆ with regard to secondary metabolism in *A. nidulans*.

Although we could not grow the *setA*∆ and H3K4R strains under same liquid AMM conditions, we were able to analyze their SM profiles from mycelia grown on solid ACM. We noticed that both strains were deeply pigmented when grown on this medium and subsequent mycelial extracts verified the accumulation of emodin and F9775A/B in both mutant strains, reminiscent of the high metabolite levels in *cclA*∆ and *kdmB*∆ grown in liquid AMM (Supplementary Figure S4). Overall, it was surprising to observe that both the lack of H3K4 methylation (*setA*∆, H3K4R, *cclA*∆) and the lack of KdmB – leading to a global increase of H3K4 methylation – result in similar phenotypes, i.e., reduced penicillin G and sterigmatocystin and at the same time strongly increased emodin and F9775A/B production.

### Histone Demethylase-Independent Role of KdmB in Secondary Metabolite Gene Regulation in *A. nidulans*

**Figure 2:**
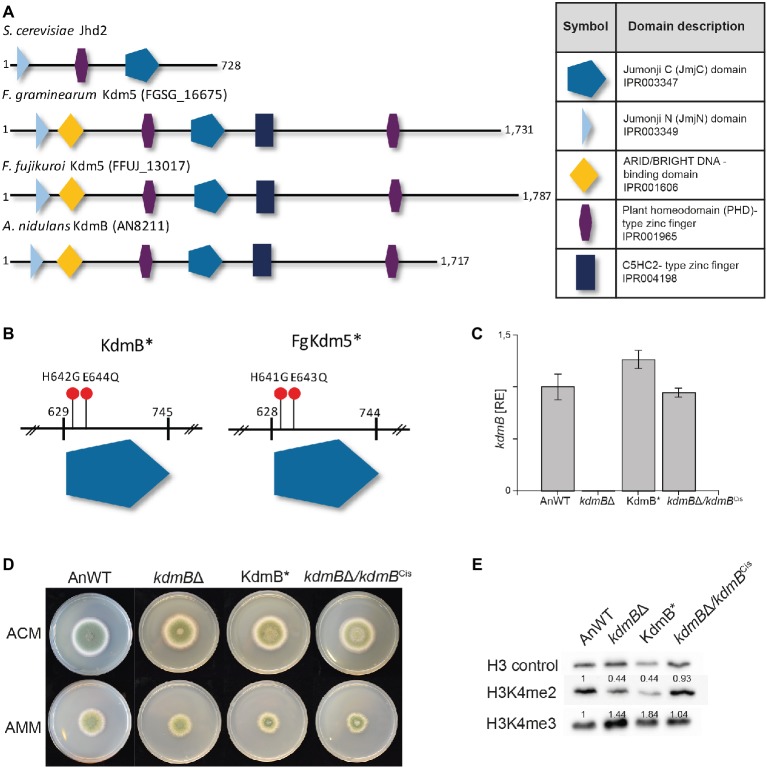
Mutation of the H3K4-specific demethylase KdmB renders the demethylase domain nonfunctional. **(A)** The conserved domain structure of KDM5 histone demethylase proteins is shown for *Saccharomyces cerevisiae*, *Fusarium graminearum, Fusarium fujikuroi,* and *Aspergillus nidulans*. **(B)** Targeted mutagenesis of the conserved JmjC-domain was accomplished by using primers harboring the desired mutation; that is H642G and E644Q for *A. nidulans*. Analogous to *A. nidulans*, the conserved amino acids important for Fe^2+^ binding and thus necessary for the integrity of the JmjC-domain are histidine 643 (H643) and glutamic acid 645 (E645) in *F. graminearum*. **(C)** The *kdmB* transcript levels of the *A. nidulans* wild-type strain (AnWT), *kdmB*Δ, KdmB*, and *kdmB*Δ/*kdmB*^Cis^ strains were measured as an additional control of generated strains. Prior to RNA extraction, indicated strains were grown in liquid AMM for 48 h. Experiments were performed in duplicates, and *kdmB* expression of AnWT was arbitrarily set as 1. Mean values and standard deviations are shown. RE, relative expression **(D)** For radial growth analysis, indicated strains were grown on solid ACM and AMM plates at 37°C under dark conditions. Pictures were taken after 5 days. Experiments were done in triplicates. Mean values and standard deviations are shown **(E)** For western blot analysis, indicated strains were grown in liquid AMM with 10 mM sodium nitrate for 48 h prior to protein extraction. Subsequently, whole protein extracts were isolated from lyophilized mycelia, and roughly, 10 μg of proteins were used for western analysis. H3 C-Term, H3K4me2, and me3 antibodies were used for detection. A densitometric analysis was performed and the protein levels of AnWT were arbitrarily set as 1. Experiments were done three times giving the same trend. Hence, only one experiment is depicted here.

Contrary to its homolog Jhd2 in *S. cerevisiae* KdmB harbors, in addition to the JmjC and JmjN domain, a second PHD-type zinc finger as well as a C5HC2-type zinc finger and an ARID/BRIGHT DNA-binding domain (Figure 2A). A similar domain structure is present in Lid2 and Lid in *Schizosaccharomyces pombe* and *D. melanogaster*, respectively. Similar to Lid, one of the PHD domains may interact with histones, but the functions and targets of other domains remain opaque (Li et al., 2010).

We wanted to define whether the demethylase activity of KdmB is required for its function in SM gene activation (PEN and ST clusters) or repression (MDP and ORS clusters). For this, we have generated a demethylase-deficient mutant by introducing two point mutations in the JmjC domain of KdmB that were previously shown in *D. melanogaster* to abolish Fe^2+^ binding in the catalytic site and consequently abrogate Lid demethylase function (Li et al., 2010). These iron-coordinating amino acids are conserved in the KdmB JmjC domain, and hence, histidine 642 was mutated to glycine (H642G) and glutamic acid 644 to glutamine (E644Q), resulting in KdmB^H642G,E644Q^, from now on designated KdmB* (Figure 2B and Supplementary Figure S5). As a control, the *kdmB*∆ was also complemented by re-introducing the fully functional *kdmB* including its native promoter and terminator sequences into the native locus resulting in *kdmB*∆/*kdmB*^Cis^ (complementation *in situ*). Several independent mutants with identical growth phenotypes were obtained in each case (Supplementary Figure S6), and subsequently, one of each was used for further analysis. RT-qPCR of *kdmB* transcript levels in both the KdmB* and the *kdmB*/*kdmB*^Cis^ verified wild type-like *kdmB* levels (Figure 2C). No significant alterations in radial hyphal growth were visible when the generated strains were grown on solid ACM or AMM (Figure 2D). Noteworthy, biomass was slightly but significantly increased to about 130% in case of both the KdmB* and the *kdmB*Δ strain when cultivated under SM-inducing conditions (liquid AMM with 10 mM sodium nitrate). The complemented *kdmB*Δ/*kdmB*^Cis^ strain showed a wild type-like growth phenotype (Supplementary Figure S7). Western blot analysis of *kdmB*Δ, KdmB*, and *kdmB*Δ/*kdmB*^Cis^ strains using anti-H3K4 methylation-specific antibodies (H3K4me2 and H3K4me3) revealed elevated H3K4me3 but reduced H3K4me2 levels in both *kdmB*Δ and KdmB* compared to AnWT and the complemented strain *kdmB*Δ/*kdmB*^Cis^. This phenotype is in accordance with our previous proteomic analyses (Gacek-Matthews et al., 2016) and likely attributable to the demethylase deficiency in both strains (Figure 2E).

**Figure 3:**
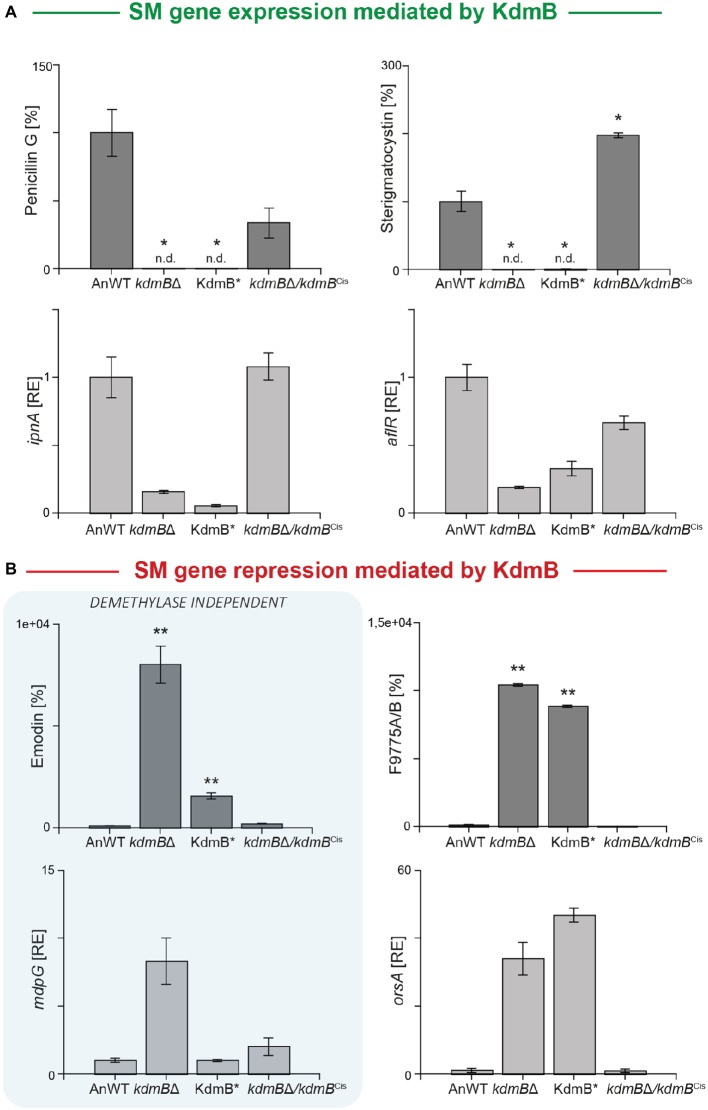
KdmB has a histone demethylase-independent role in regulation of the emodin gene cluster. **(A,B)** For SM analysis, the *A. nidulans* wild-type strain (AnWT), *kdmB*Δ, KdmB*, and *kdmB*Δ/*kdmB*^Cis^ strains were grown in liquid AMM with 10 mM sodium nitrate for 48°h. Supernatants were applied for HPLC-MS/MS measurements. To exclude falsifications in SM biosynthesis due to differences in hyphal growth, SM production was correlated to biomass formation. For a direct comparison, production of the wild type was arbitrarily set to 100%. For statistical analysis, a student’s *t* test was performed: ^*^p < 0.05; ^**^p < 0.01; n.d., not detected. For determination of transcript levels, RNA was extracted of 48 h old, lyophilized mycelia. The transcription levels of *ipnA* [encoding the non-ribosomal peptide synthetase (NRPS) required for penicillin (PEN) production], *aflR* [encoding the pathway-specific transcription factor for sterigmatocystin (ST) biosynthesis], *orsA* [encoding a non-reducing polyketide synthase (NR-PKS) required for orsellinic acid and F9775A/B biosynthesis], and *mdpG* (encoding a NR-PKS required for emodin biosynthesis) were determined *via* RT-qPCR. SM data represent metabolite accumulation over time, whereas gene transcription was measured at a definite time point (*ipnA, aflR, mdpG*: 48 h; *orsA*: 24 h). Expression level of AnWT was arbitrarily set as 1 for the individual SM genes. Experiments were done in triplicates. Mean values and standard deviations are shown. RE, relative expression.

We used these strains now to gain a deeper insight into the relevance of the KdmB histone demethylase function for SM gene regulation and cultivated them for 48 h under SM-inducing conditions (AMM with 10 mM sodium nitrate). For subsequent SM quantification, we focused on those clusters that have been identified as being KdmB- and H3K4 methylation-dependent in their activation (penicillin G and sterigmatocystin), or repression (emodin and F9775A/B). Surprisingly, both activated clusters remained silent not only in the *kdmB*Δ mutant but also in the KdmB* strain indicating that the full integrity of the JmjC demethylase domain with iron-coordinating H642 and E644 residues is crucial for the activating function of KdmB (Figure 3A). However, in respect to the repressing function of KdmB, we found that only the repression of F9775A/B biosynthesis was dependent on these conserved amino acids. Emodin, on the contrary, was not affected. The strain harboring the mutated KdmB (KdmB*) produced very low levels of this metabolite and showed only background expression of the biosynthetic gene (*mdpG*), similar to the *A. nidulans* wild-type control (Figure 3B). Taken together, KdmB functions in gene induction (penicillin G and sterigmatocystin) as well as repression (F9775A/B) in a strictly histone demethylase-dependent manner, while the repression of emodin biosynthesis appears to depend on KdmB but is independent of its function as a histone demethylase.

### The Histone Demethylase-Independent Role in Secondary Metabolite Gene Regulation Is Conserved Also in the Phytopathogenic Fungus *Fusarium graminearum*

KDM5 proteins belong to the diverse JmjC domain-containing superfamily, which have been identified in all living organisms from bacteria to higher eukaryotes and are characterized by the highly conserved JmjC domain (Accari and Fisher, 2015). Thus, we next analyzed whether the function of the histone demethylase and the histone demethylase-independent function of KdmB* is conserved in other filamentous fungi. For this, we identified and deleted as well as mutated the KDM5 homolog designated FgKdm5, in the wheat pathogen *F. graminearum* PH-1 wild-type strain (FgWT). Three independent Δ*fgkdm5* mutant strains were produced, and full deletion was verified by diagnostic PCR and Southern blot (Supplementary Figures S8A,B,C). Next to ∆*fgkdm5*, the JmjC domain of FgKdm5 was mutated at equivalent positions like the *A. nidulans* KdmB*, i.e., histidine 641 was mutated to glycine (H641G) and glutamic acid 643 to glutamine (E643Q). Out of this, three independent FgKdm5^H641G,E643Q^ strains were obtained, hereafter referred to as FgKdm5*. Successful generation of both mutant strains was subsequently verified by diagnostic PCR (Supplementary Figures S8D,E). In addition, the native *FgKDM5* was reintroduced into ∆*fgkdm5* in order to complement the loss of *FgKDM5*. Notably, *in situ* integration was unsuccessful so far. Therefore, *FgKDM5* was introduced heterologously at a random location in the genome (*ex situ*), but expression was still driven by its native promoter and terminator sequences. Obtained mutants designated Δ*fgkdm5/FgKDM5*^Ces^ (complementation *ex situ*) were verified by diagnostic PCR (Supplementary Figures S8F,G) and transcription of *FgKDM5* tested by RT-qPCR. This analysis verified functional integration of the complementing gene as wild type-like *FgKDM5* levels were detected in both FgKdm5* and Δ*fgkdm5/FgKDM5*^Ces^, while the transcript was absent from the Δ*fgkdm5* strain (Supplementary Figure S9A). Western blot analysis using anti-H3K4 methylation-specific antibodies for mono-, di-, and trimethylation confirmed the non-functional demethylase domain of FgKdm5* as we observed increased H3K4 tri- and reduced dimethylation levels in the mutant protein extracts compared to FgWT (Supplementary Figure S9B). H3K4 di- and trimethylation levels were restored to FgWT levels in the complemented Δ*fgkdm5/FgKDM5*^Ces^ strain (Supplementary Figure S9B).

**Figure 4:**
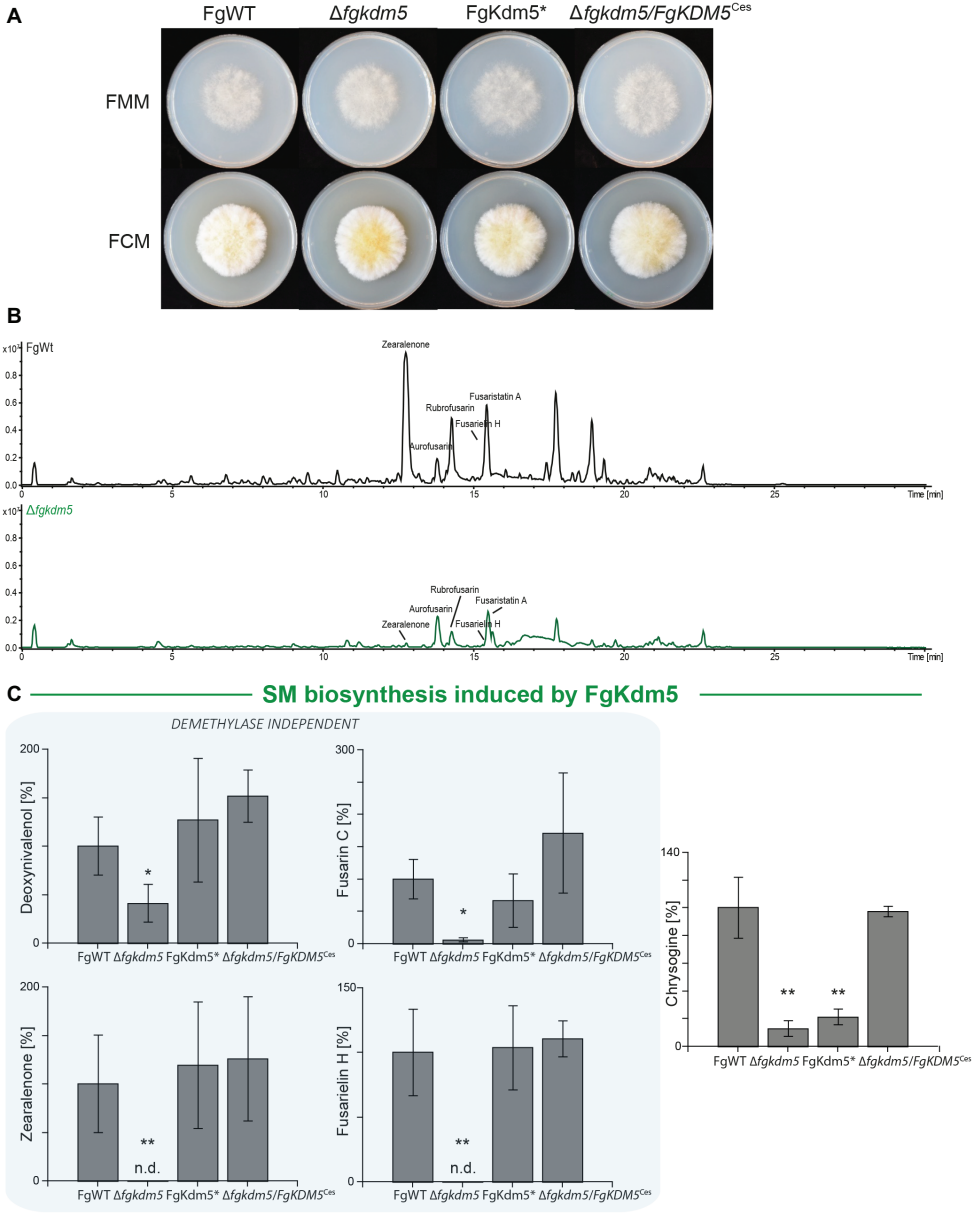
Kdm5 is also relevant for SM gene cluster expression in *Fusarium graminearum*. **(A)** For radial growth analysis, the *F. graminearum* WT (FgWT) strain, Δ*fgkdm5*, FgKdm5*, and Δ*fgkdm5/FgKDM5*^Ces^ strains were grown on solid FCM and FMM plates at 20°C. Pictures were taken 5-day post inoculation. **(B-C)** For SM analysis, the indicated strains were grown on PDA plates for 2 weeks at 20°C. Subsequently, agar plugs were extracted and applied for HPLC-MS/MS analysis. The corresponding chromatogram is shown for FgWT and Δ*fgkdm5*. Experiments were performed in biological triplicates with three independent mutants and SM production of FgWT was arbitrarily set to 100%. Mean values and standard deviations are shown. For statistical analysis, a student’s *t* test was performed: ^*^p < 0.05; ^**^p < 0.01; n.d., not detected.

Similar to the observations in *A. nidulans*, hyphal growth of ∆*fgkdm5* and FgKdm5* was not significantly altered when grown on solid Fusarium minimal or complete medium, FMM and FCM, respectively (Figure 4A and Supplementary Figure S9C). We also tested the involvement of FgKdm5 and its demethylase domain in the production of SMs. It is well known that *F. graminearum* sports a great variety of these compounds. 67 putative gene clusters have been predicted to be involved in SM biosynthesis (Sieber et al., 2014). Next to aurofusarin (Kim et al., 2005; Malz et al., 2005), deoxynivalenol (Kogel et al., 2005; Life, 2006; Audenaert et al., 2013), zearalenone (Lysøe et al., 2006), and fusarin C (Farber and Sanders, 1986), several novel products as fusarielins (Sørensen et al., 2014; Wollenberg et al., 2016), fusaristatins (Sørensen et al., 2014), and chrysogine (Wollenberg et al., 2017) have been characterized in *F. graminearum.* To analyze changes in secondary metabolism, generated strains were grown together with FgWT for 2 weeks on potato dextrose agar (PDA) as described by Giese et al. (2013). Similar to *A. nidulans* and *F. fujikuroi* (Gacek-Matthews et al., 2016; Janevska et al., 2018), deletion of *FgKDM5* resulted in a significant decrease of several SMs (Figure 4B). More specifically, production of the mycotoxins deoxynivalenol and fusarin C, the mycoestrogens zearalenone and fusarielin H as well as production of chrysogine was significantly decreased in strains lacking FgKdm5, while aurofusarin and fusaristatin A levels remained unaffected (Figure 4C and Supplementary Figure S10). To our surprise, most of the SMs affected by Δ*fgkdm5* appeared to be regulated by FgKdm5 independently of the integrity of the JmjC domain and its putative histone demethylase function as deoxynivalenol, zearalenone, fusarin C, and fusarielin H production levels are reaching FgWT levels in the FgKdm5* strain (Figure 4C). The only SM analyzed here which biosynthesis required the Fe^2+^-coordinating residues was chrysogine. Chrysogine was decreased in both, *∆fgkdm5* and FgKdm5* strains (Figure 4C). As expected, production level of aurofusarin and fusaristatin A that were not affected in Δ*fgkdm5* remained unchanged also in FgKdm5* (Supplementary Figure S10). Complementation of ∆*fgkdm5* with the native *FgKDM5,* i.e., ∆*fgkdm5/FgKDM5*^Ces^ restored productions levels to FgWT (Figure 4C). Taken together, several SMs in *F. graminearum* depend on FgKdm5 but unlike KdmB in *A. nidulans*, the JmjC domain of FgKdm5 is not crucial for this activation function. Only in case of chrysogine, the histone demethylase function of FgKdm5 is essential.

### *kdmB* Cannot Rescue the Demethylase-Dependent Phenotype in *∆fgkdm5*

**Figure 5:**
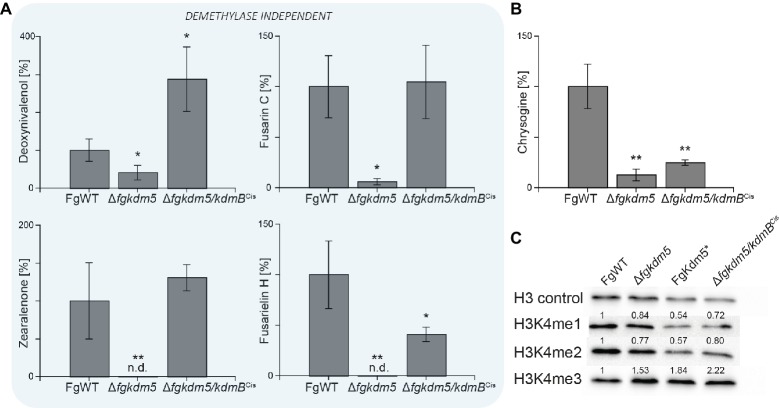
*kdmB* cannot rescue the demethylase-dependent phenotype in ∆*fgkdm5.* The *F. graminearum* WT (FgWT), Δ*fgkdm5*, FgKdm5*, and Δ*fgkdm5/kdmB*^Cis^ were grown on PDA plates at 20°C for 2 weeks for **(A)** deoxynivalenol, fusarin C, zearalenone, fusarielin H, and **(B)** chrysogine production. Subsequently, agar plugs were extracted and applied for HPLC-MS/MS analysis. Experiments were performed in triplicates with three independent mutants, and SM production of FgWT was arbitrarily set to 100%. Mean values and standard deviations are given. For statistical analysis, a student’s *t* test was performed: ^*^p < 0.05; ^**^p < 0.01; n.d., not detected. **(C)** For western blot analysis FgWT, Δ*fgkdm5*, FgKdm5*, and Δ*fgkdm5/kdmB*^Cis^ strains were grown for 3 days on solid complete medium. Whole protein extracts were subsequently isolated from lyophilized mycelia, and roughly, 15 μg of proteins were used for SDS-Page and western blotting. H3 C-Term, H3K4me1, H3K4me2, and H3K4me3 antibodies were used for detection. A densitometric analysis was performed, and FgWT was arbitrarily set to 1. Experiments were performed three times giving the same trend. Hence, only one experiment is depicted here.

Next, we analyzed if *kdmB* of *A. nidulans* mediates activation of SMs in *F. graminearum* and if the integrity of the KdmB JmjC domain is necessary for this activation function. To test this, the native *kdmB* was amplified from *A. nidulans* genomic DNA and sequence-verified plasmids integrated into the native *FgKDM5* locus in *F. graminearum*. This resulted in ∆*fgkdm5/kdmB*^Cis^ (complementation *in situ*) in which *kdmB* is driven by the *F. graminearum* promoter (Supplementary Figures S11A,B). Subsequent RT-qPCR of the *kdmB* transcript in three independent ∆*fgkdm5/kdmB*^Cis^ mutants verified successful expression of *kdmB* (Supplementary Figure S11C). As expected and similar to ∆*fgkdm5*, the ∆*fgkdm5/kdmB*^Cis^ strains did not show an altered growth phenotype compared to FgWT when grown on solid FCM or FMM (Supplementary Figures S12A,B). To test whether *kdmB* can complement for the loss of *FgKDM5* with regard to secondary metabolism, FgWT, Δ*fgkdm5* and ∆*fgkdm5/kdmB*^Cis^ strains were grown for up to 2 weeks on PDA. The ∆*fgkdm5/kdmB*^Cis^ strain rescued SM levels in case of deoxynivalenol, zearalenone, fusarin C and fusarielin H, while chrysogine levels were still reduced in the ∆*fgkdm5/kdmB*^Cis^ like in the Δ*fgkdm5* strain (Figure 5A). It is noteworthy that chrysogine appeared to be the only SM analyzed here that strictly depended on a functional histone demethylase domain (Figure 5B), suggesting that *kdmB* cannot rescue the histone demethylase function in ∆*fgkdm5*. Intrigued by these findings, we next performed western blot experiments using anti-H3K4me3-specific antibodies. Indeed, H3K4me3 levels are similarly increased in ∆*fgkdm5/kdmB*^Cis^ as compared to Δ*fgkdm5*, while the complementation with the native gene (Δ*fgkdm5/FgKDM5*^Ces^) resulted in H3K4me3 that were comparable to FgWT (Figure 5C). Thus, cross-complementation experiments where ∆*fgkdm5* is complemented with *kdmB* suggest that *kdmB* can complement the histone demethylase-independent functions of FgKdm5 in SM gene regulation but not JmjC-dependent functions.

## Discussion

H3K4me3 is a well-studied chromatin mark involved not only in transcriptional initiation and elongation but also in recombination site selection during meiosis (Acquaviva et al., 2013; Adam et al., 2018). H3K4me3 are found predominantly at transcriptional start sites of transcribed genes (Santos-Rosa et al., 2002; Soares et al., 2017). Consequently, proteins involved in writing and erasing this histone PTM are associated with transcriptional activation and repression, respectively. H3K4me is established and erased by members of COMPASS/Set1 and KDM5, respectively. The latter belong to the Jumonji domain proteins of the KDM5 family and are involved in recognition and removal of H3K4me3 marks and thus resulting typically in repression of the targeted loci (Klose et al., 2006, 2007; Seward et al., 2007; Thinnes et al., 2014). However, these proteins consist of several domains that might be important for their diverse functions in chromatin and transcriptional regulation.

Previously we could show that KdmB is mediating not only transcriptional silencing but also transcriptional activation of SMGCs in *A. nidulans* (Gacek-Matthews et al., 2016). In detail, KdmB serves as a canonical, transcriptional repressor for the SMGCs monodictyphenone/emodin, and F9775A/B in *A. nidulans*. Strikingly, our data show that only for mediating repression of the F9775A/B gene cluster the integrity of the JmjC domain is important. Contrary to this, emodin biosynthesis and *mdpG* expression did not depend on a functional JmjC domain as KdmB* mostly phenocopied AnWT. Demethylase-independent functions of KDM5 proteins have been described, but mostly in context with gene activation. For example, studies in *D. melanogaster* showed that loss of the essential KDM5 homolog Lid (*little imaginal discs*) results in retarded larval development (Li et al., 2010), and this delay is independent of the histone demethylase activity encoded by the JmjC domain, suggesting that other domains of Lid are critical during early developmental processes (Drelon et al., 2018). Furthermore, functions in gene regulation that are independent of the catalytic activity are not restricted to KDM5 homologs or histone demethylases. Thus, demethylase-independent regulations have also been described for the Lysine Specific Demethylase 1 LSD1 in human cancer cells. LSD1 promotes the survival of prostate cancer cells independently of its demethylase function (Sehrawat et al., 2018). Also, the methyltransferase Set1 in *S. cerevisiae* exhibits chromatin regulatory functions independent of H3K4 methylation (Lee et al., 2018). Here, the enzymatic activity of Set1 might be directed to a non-H3K4 substrate, which in turn has chromatin-associated regulatory functions (Mikheyeva et al., 2014). Thus, it might be possible that in our case KdmB utilizes a different domain to repress the emodin gene cluster in *A. nidulans*. However, strains largely lacking H3K4 tri-, di-, and monomethylation (*setA*∆, H3K4R and *cclA*∆) as well as loss of KdmB show overlapping phenotypes with regard to emodin and F9775A/B biosynthesis. Nevertheless, the increase of both SMs was still significantly lower in *kdmB*∆ as compared to *cclA*∆, suggesting that KdmB is part of a repression complex that requires H3K4me3 or me2 and that these marks are crucial for mediating repression. Whether the repressive function of KdmB is mediated directly at these loci and *via* H3K4me-binding or happens through the recruitment of different *trans*-factors (e.g., deacetylases) still remains to be elucidated. So far, we could not find any repressing function of FgKdm5 in *F. graminearum* regarding the SMs analyzed in this study. This is in agreement with recently published data in *F. fujikuroi*, where all SMs analyzed were found to be decreased by deletion of *FfKDM5* (Janevska et al., 2018). However, a genome-wide transcriptional analysis in this fungus showed that FfKdm5 also functions in SM gene repression although to a lesser extent. Thus, it is likely that some SMGCs are repressed by FgKdm5 also in *F. graminearum* similar to *F. fujikuroi* and *A. nidulans*.

Activating functions of KdmB were found at the penicillin G and sterigmatocystin biosynthesis loci in *A. nidulans*. In these cases, production of both SMs strictly depends on a functional histone demethylase-domain of KdmB. Additionally, our data demonstrate that H3K4 methylation is needed at these gene loci for activation as *cclA*∆ largely phenocopies the loss of KdmB. This SM-activating function is conserved also in *F. graminearum*. In fact, from the analyzed SMs that are affected by the loss of FgKdm5, all of them were found to be downregulated in ∆*fgkdm5*. Similarly, lack of the KDM5 homolog KdmB in *Epichloë festucae* resulted in reduced expression of the SMGCs involved in lolitrem (*ltm*) as well as ergoalkaloids and indole diterpenes (*eas*) biosynthesis (Lukito et al., 2019). Contrary to *A. nidulans* but in line with *F. fujikuroi* (Janevska et al., 2018), *Fusarium* strains deficient for H3K4 methylation did not phenocopy the ∆*fgkdm5* strain. More specifically, only production of the virulence factor deoxynivalenol was found to be regulated in a similar manner in ∆*fgkdm5* and ∆*fgccl1*, while fusarin and zearalenone biosynthesis were regulated antagonistically in the two strains [this study; Studt et al., 2017)]. It is noteworthy that the same goes true for *F. fujikuroi*. Here, ∆*ffkdm5* and ∆*ffccl1* both resulted in reduced levels of gibberellic acid (Studt et al., 2017; Janevska et al., 2018), the virulence factor that facilitates colonization of rice roots by this fungus (Wiemann et al., 2013). Aurofusarin biosynthesis remained unaffected in both ∆*fgkdm5* and ∆*fgccl1.*

The obvious difference between *cclA*∆ on the one hand and ∆*fgccl* and ∆*ffccl1* on the other hand is the remaining H3K4 methylation levels. While hardly any H3K4 mono-, di-, and trimethylation is detectable in *cclA*∆, only H3K4 trimethylation is affected in both ∆*fgccl* and ∆*ffccl1* (Studt et al., 2017). The latter being in agreement with strains deleted for *cclA* in *E. festucae* that showed reduced but not abolished H3K4me3 levels (Lukito et al., 2019). Here, the *LTM* and *EAS* cluster genes are silenced by CclA. Notably, both SMGCs reside in subtelomeric regions. It is intriguing to speculate that the distinct H3K4 methylation levels account for the observed differences in the phenotypes with regard to *kdmB*∆ as well as *∆fgkdm5* and *∆ffkdm5*. Surprisingly and again contrary to our data in *A. nidulans*, FgKdm5 activates the majority of the analyzed SMGCs (deoxynivalenol, zearalenone, fusarin C, and fusarielin H) independently of the demethylase-domain. Only the SMGC involved in chrysogine biosynthesis seems to be dependent on the functional JmjC domain of FgKdm5. Thus, it is possible that the activating functions of KDM5-proteins are conserved between *A. nidulans* and *F. graminearum*, but different domains are utilized for this task. In respect to SM regulation, the activating H3K4me3 histone mark is largely absent from most of the SMGCs in *A. nidulans* (Gacek-Matthews et al., 2016) (Supplementary Figure S13). The same is true for *F. graminearum* (Connolly et al., 2013) and *F. fujikuroi* (Wiemann et al., 2013; Studt et al., 2017; Janevska et al., 2018). These data would be consistent with two interpretations of SMGC regulation by KDM5 family members: either KDM5 homologs function in regulating SMGCs indirectly *via trans*-acting factors and/or by utilizing different domains for activating or repressing targeted SMGCs. Next to JmjC, Kdm5 harbors five further putative domains: a JmjN (IPR003349), an AT-rich interaction domain (ARID, IPR001606), two plant homeodomain (PHD) – type zinc fingers (IPR001965) and a C5HC2- type zinc finger (IPR004198). Interestingly, similar KDM5-domain studies in *D. melanogaster* revealed that activation of essential mitochondrial genes happens independently of the JmjC demethylase activity but relies on the C-terminal PHD domain that binds to H3K4me2/3 (Liu and Secombe, 2015). Thus, it would be interesting to see whether one of the aforementioned domains is involved in direct targeting and/or binding. Mutation or deletion of single domains will show if one of them is required for transcriptional regulation in the future.

Cross-complementation experiments revealed that KdmB could only partially complement the ∆*fgkdm5* phenotype. Global H3K4me3 levels were enriched in both ∆*fgkdm5*/*kdmB*^Cis^ and ∆*fgkdm5.* In agreement with this, chrysogine levels – the only SM analyzed in *F. graminearum* that was shown to strictly depend on the FgKdm5 demethylase function – were similarly decreased in both ∆*fgkdm5*/*kdmB*^Cis^ and ∆*fgkdm5*. By contrast, all SMs that are regulated independently of the demethylase function were fully restored to FgWT levels in a ∆*fgkdm5*/*kdmB*^Cis^ strain. Notably, although domain synteny is conserved, KdmB and FgKdm5 exhibited a rather low amino acid sequence identity of only 58%. Amino acid sequence analysis of the different domains revealed quite high identities for the JmjC (87%), ARID/Bright (86%), and the C-terminal PHD (82%) domain, whereas rather low identities were gained for the JmjN (69%), the N-terminal PHD (55%) domain, and the C5HC2 type zinc finger (41%). Thus, it is surprising that it is the catalytic function of the JmjC domain that is not rescued by KdmB in a ∆*fgkdm5* strain, while demethylase-unrelated phenotypes are restored in the cross-complementation approach with regard to SM gene regulation. Thus, it would be interesting to analyze whether KDM5 orthologs from other fungal species are able to functionally complement the histone demethylase domain in ∆*fgkdm5*. In a similar study, several orthologs from different distantly related Ascomycetes, i.e., *A. nidulans* (Eurotiomycetes), *Alternaria alternata* (Dothideomycetes), *F. fujikuroi* (Sordariomycetes), and from the close relative *Sclerotinia sclerotiorum* (Leotiomycetes), failed to functionally replace the H3K36-specific demethylase (∆*bckdm1*) in *Botrytis cinerea* (Schumacher et al., 2019). Similar to our study, the proteins exhibited overall low sequence conservation, e.g., the sequence identity between KdmA and BcKdm1 was only 31.1%. However, it remains to be seen whether this is due to a non-functioning histone demethylase as observed for KdmB expressed in the ∆*fgkdm5* deletion strain.

Overall, this study demonstrates the complex regulatory circuits of KDM5 homologs in the two Ascomycetes *A. nidulans* and *F. graminearum*. In both organisms, a functional homolog of KDM5, i.e., KdmB and FgKdm5, respectively, is required for wild type-like SM gene regulation Interestingly, the integrity of the JmjC domain is not always essential for mediating repression or activation of specific SMGCs. At which loci, KdmB/FgKdm5 function mediated indirectly *via* targeting H3K4me3 mainly present outside SMGCs and/or directly by interacting with chromatin complexes present at the regulated SMGCs remains to be elucidated. Future ChIP-seq analyses using tagged KDM5 proteins will allow us to map their genomic positions under different metabolic conditions and thereby help us to understand their bipartite function in activation and repression of SMGCs mechanistically.

## Data Availability

The datasets generated for this study are available on request to the corresponding author.

## Author Contributions

SB, LS, and JS contributed to the design of the work. SB, JLS, MS, and AG-M were involved in data acquisition. SB, LS, JS, JLS, MS, and AG-M were involved in data analysis. SB, LS, and JS wrote the manuscript. All authors revised and approved the manuscript.

### Conflict of Interest Statement

The authors declare that the research was conducted in the absence of any commercial or financial relationships that could be construed as a potential conflict of interest.
