# Supplementary material for: Evidence of a Demethylase-Independent Role for the H3K4-Specific Histone Demethylases in Aspergillus nidulans and Fusarium graminearum Secondary Metabolism
Source: Front Microbiol. 2019 Aug 13;10:1759. doi: 10.3389/fmicb.2019.01759 (PMC6700381; doi:10.3389/fmicb.2019.01759)
Supplement: Supplementary file 1 [file Table_1.DOCX]

Supplementary Material

**Evidence of a demethylase-independent role of the H3K4-specific histone demethylases in *Aspergillus nidulans* and *Fusarium graminearum* secondary metabolism**

**Simone Bachleitner^1^, Jens Laurids Sørensen^2^, Agnieszka Gacek-Matthews^1#^, Michael Sulyok^3^, Lena Studt^1*^ and Joseph Strauss^1*^**

^1^ Department of Applied Genetics and Cell Biology, University of Natural Resources and Life Sciences, Vienna (BOKU), Konrad-Lorenz-Straße 24, 3430 Tulln an der Donau, Austria

^2^ Department of Biotechnology, Chemistry and Environmental Engineering, Aalborg University, DK-9000 Aalborg, Denmark

^3^ Institute of Bioanalytics and Agro-Metabolomics, Department for Agrobiotechnology (IFA-Tulln), University of Natural Resources and Life Sciences, Vienna (BOKU), Konrad-Lorenz-Str. 20, Tulln 3430, Austria;

^#^current address: Institute of Microbiology, University of Veterinary Medicine, Vienna, Austria;

*** Correspondence:**

Dr. Joseph Strauss

[joseph.strauss@boku.ac.at](mailto:joseph.strauss@boku.ac.at)

Dr. Lena Studt

[lena.studt@boku.ac.at](mailto:lena.studt@boku.ac.at)

**Figure S1**


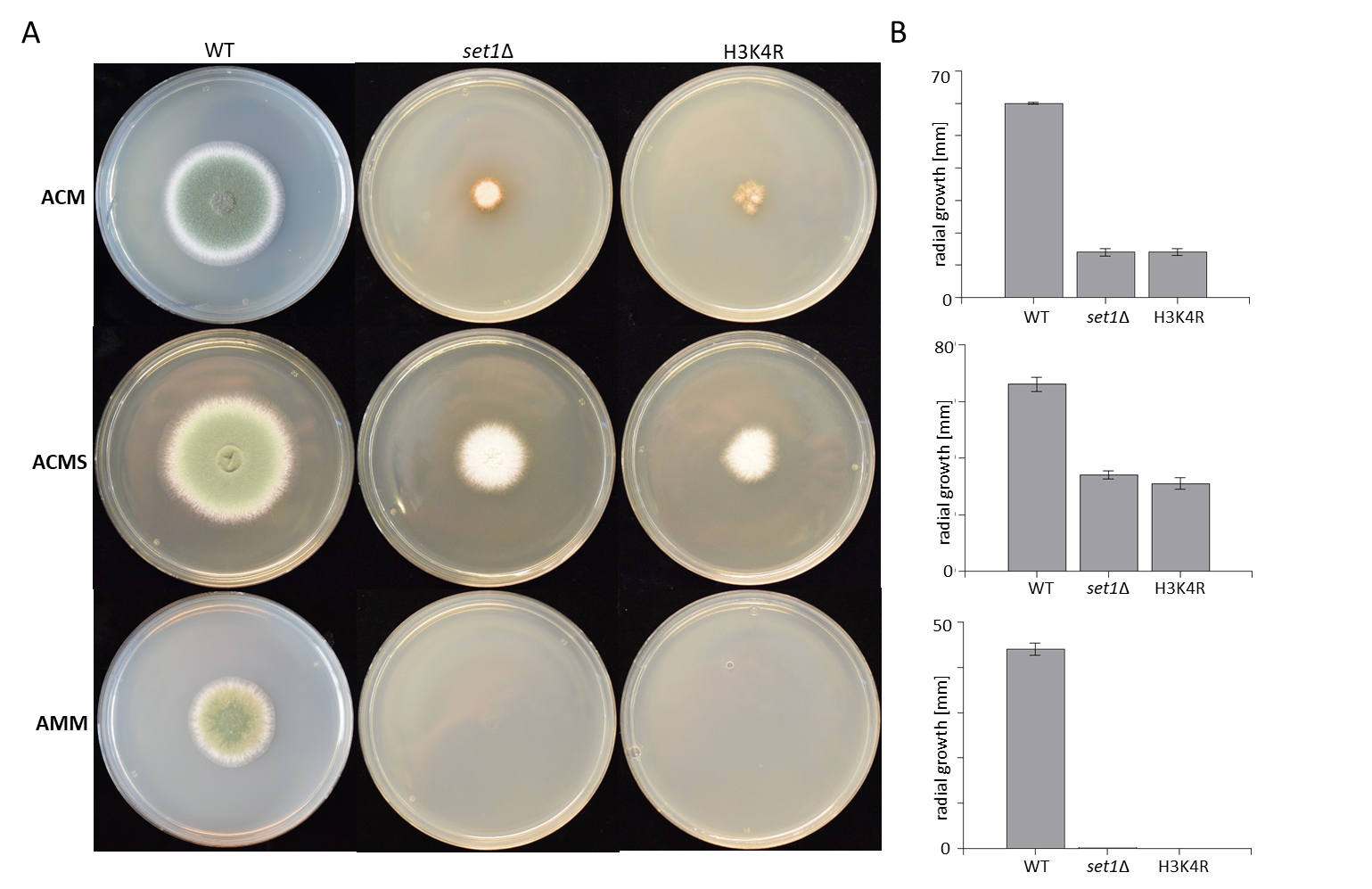


**Figure S1. Mutants deficient in H3K4me1, -me2 and -me3 (*set1*Δ, H3K4R) show a crippled growth phenotype. (A-B)** The *Aspergillus nidulans* wild-type strain (AnWT), the *set1*∆ and the H3K4R strain were grown on Aspergillus minimal medium (AMM), complete medium (ACM) and ACM supplemented with 1 M sorbitol (ACMS) at 37°C in the dark. Pictures and radial, hyphal growth were assessed 5 days post inoculation.

**Figure S2**


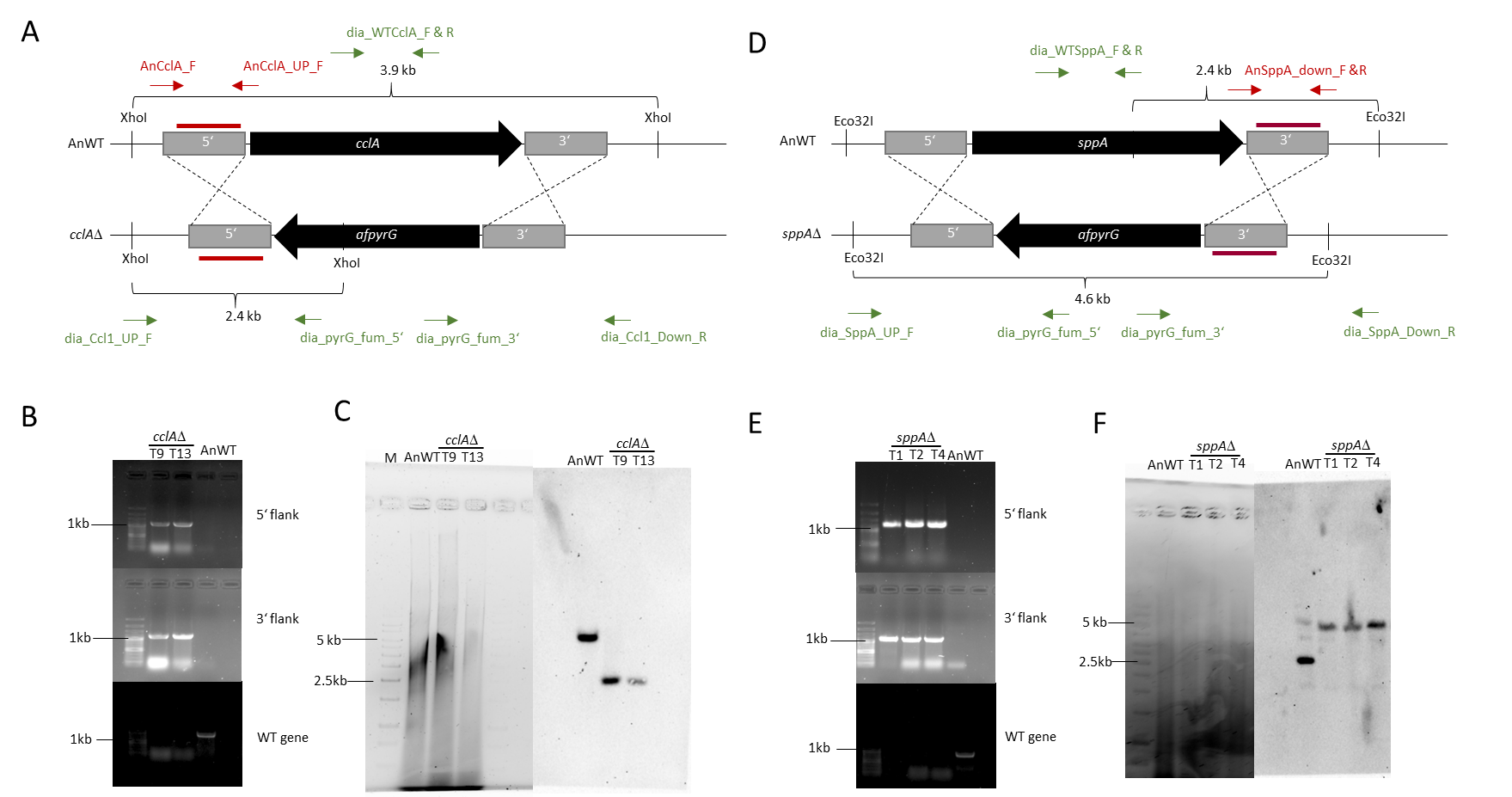


**Figure S2. Verification of *Aspergillus nidulans* *cclA*Δ and *sppA*Δ deletion mutants by diagnostic PCR and Southern blot.** **(A)** Deletion and verification strategy of *cclA*Δ mutants in *A. nidulans*. Primers for diagnostic PCR are indicated in green. The probe (red bar) for excluding ectopic integration events of the deletion construct by Southern blot was amplified with the primers AnCclA_F//AnCclA_UP_R. Primers are indicated in red. *Xho*I was used for genomic restriction digest. **(B)**Deletion *via* homologous recombination with the *A. fumigatus pyrG* selection marker (*afpyrG*) was verified by presence of 5’ (dia_Ccl1_UP_F//dia_pyrG_fum_5’) and 3’ (dia_Ccl1_Down_R//dia_pyrG_fum_3’) flanks as well as absence of the wild-type gene (dia_WTCclA_F//dia_WT_CclA_R) for two independent transformants *cclA*Δ T9 and *cclA*Δ T13. Genomic DNA of *A. nidulans* wild type (AnWT) served as control. **C)** Southern blotting of *Xho*I-restricted genomic DNA of *cclA*Δ transformants and AnWT. The 5’ flank was used for probing as indicted (A). Detected signals match the expected 3.9 kb for AnWT and 2.4 kb for the *cclA*Δ mutants. AnWT served as control. **D)** Deletion and verification strategy of *sppA*Δ mutants in *A. nidulans.* Primers for diagnostic PCR are indicated in green. The probe (red bar) for excluding ectopic integration events was amplified with the primer pair An_SppA_Down_F//An_SppA_Down_R (indicated in red). *Eco*32I was used for genomic restriction digest. **E)** Deletion *via* homologous recombination with the *afpyrG* selection marker was verified by presence of 5’ (dia_SppA_UP_F//dia_pyrG_fum_5’) and 3’ (dia_SppA_Down_R//dia_pyrG_fum_3’) flanks as well as absence of the wild-type gene (dia_WTSppA_F//dia_WTSppA_R) for three independent transformants *sppA*Δ T1, *sppA*Δ T2 and *sppA*Δ T4. Genomic DNA of AnWT served as control.**F)** Southern blotting of *Eco*32I-restricted genomic DNA of transformants and AnWT. The 3’ flank was used for probing. Detected signals match the expected 2.4 kb for AnWT and 4.6 kb for the *sppA*Δ mutants. M, GeneRuler 1 kb DNA Ladder, Thermo Fisher Scientific.

**Figure S3**


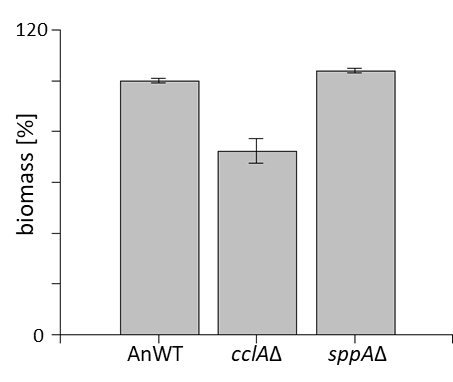


**Figure S3. Biomass formation of *Aspergillus nidulans* *cclA*Δ and *sppA*Δ mutants in liquid AMM cultures. The *A. nidulans* wild type (AnWT) as well as the *cclA*∆ and *sppA*∆ mutants**  were grown in liquid AMM with 10 mM sodium nitrate for 48 h at 37°C, 180 rpm in the dark. Subsequently, mycelia was harvested and lyophilized prior to biomass determination. The experiment was performed in biological and technical duplicates (only one of the the two biological repeats is depicted here). For comparison, biomass of AnWT was arbitrarily set to 100 %. Mean values and standard deviations are shown.

**Figure S4**


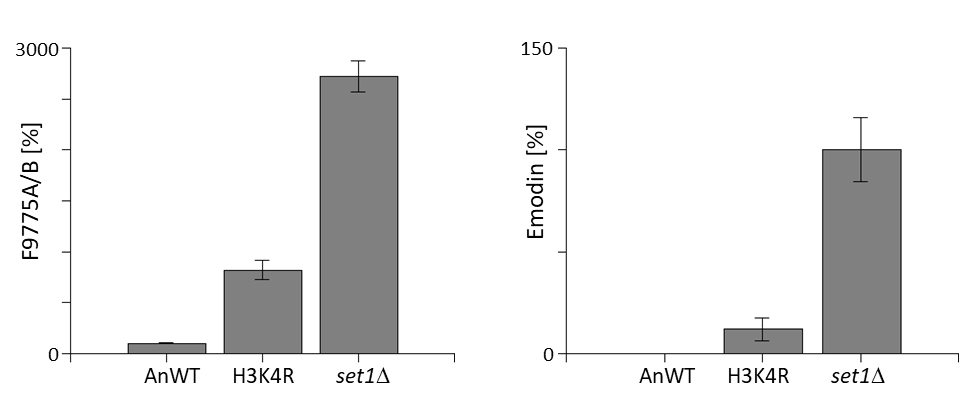


**Figure S4.Strains deficient in H3K4me1, -me2 and -me3 produce higher amounts of F9775A/B and emodin compared to the *Aspergillus nidulans* wild-type strain (AnWT).** The AnWT, the H3K4R and the *set1*∆ strains were grown on ACM at 37°C until a brownish coloring of the media appeared (approximately after 2 weeks). Subsequently, secondary metabolites were extracted out of agar plug and analyzed by HPLC-MS/MS. The experiments were performed in technical triplicates. For comparison, either production of AnWT (F9775A/B) or of *set1*∆ (emodin) were arbitrarily set to 100 %. Mean values and standard deviations are shown.

**Figure S5**


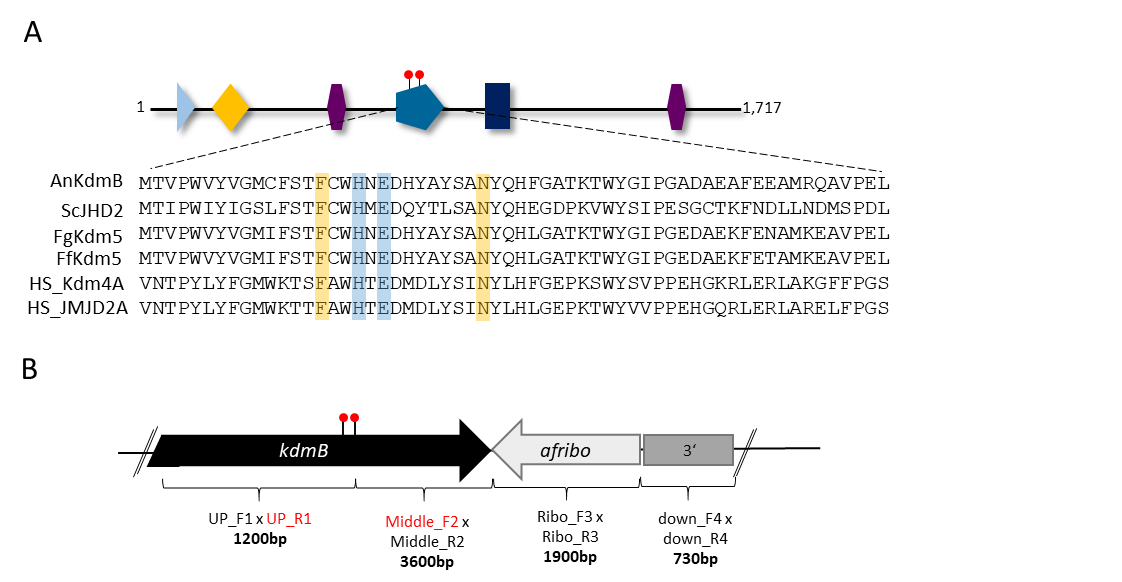


**Fig. S5. Alignment of the JmjC domain and mutation strategy of KdmB**. **(A)** For multiple sequence alignment of JmjC domain containing proteins, the protein sequences of the respective orthologues of *Aspergillus nidulans* (AnKdmB), *Saccharomycese cerevisiae* (ScJHD2), *Fusarium graminearum* (FgKdm5), *Fusarium fujikuroi* (FfKdm5) and *Homo sapiens* (HS_Kdm4A, HS_JMJD2A) were used. Conserved amino acids important for Fe^2+^ binding and thus for correct functioning of the demethylase domain are highlighted in blue (*A. nidulans* H642 and E644) whereas residues necessary for α-ketoglutarate cofactor binding (*A. nidulans* F639, N651) are highlighted in yellow. **(B)** Targeted mutagenesis was performed by using primers harboring the desired mutations (indicated in red). H642 and E644 were mutated into glycine and glutamine, respectively.

**Figure S6**


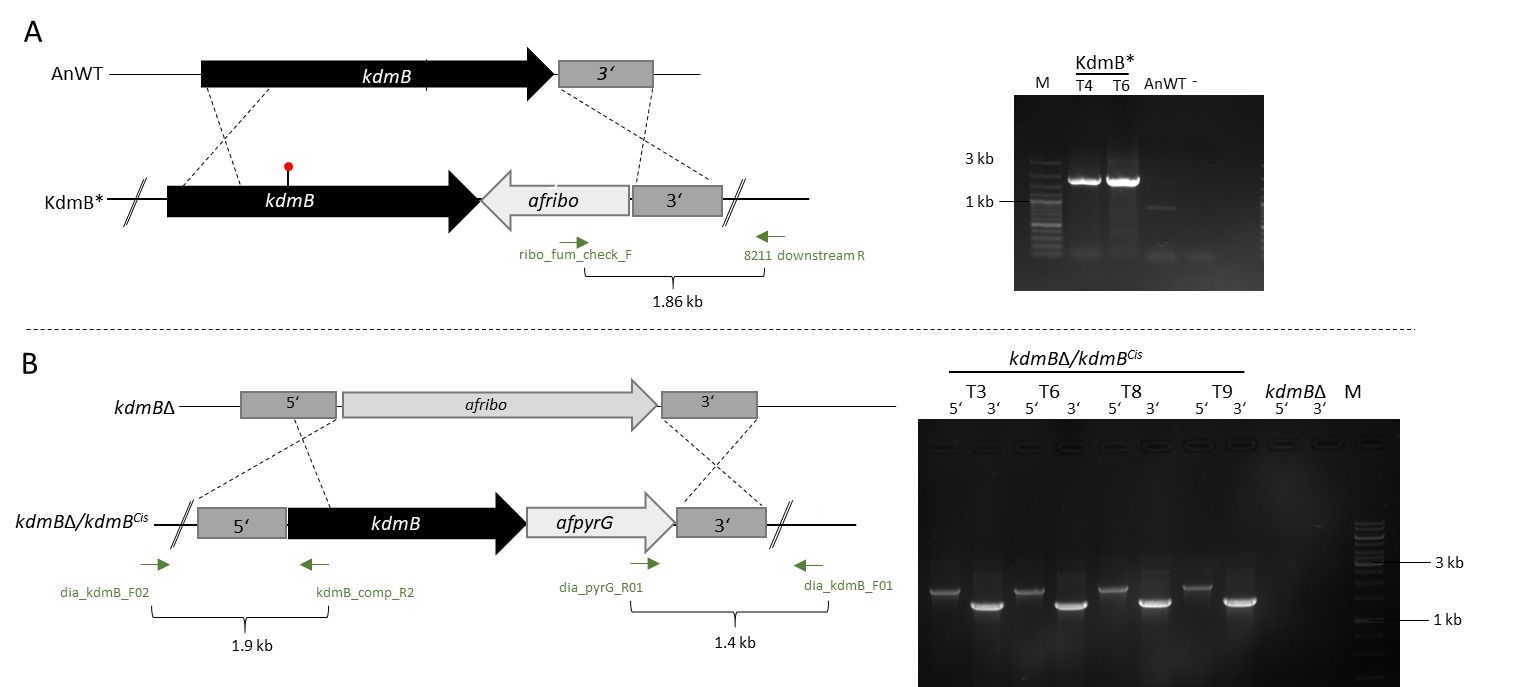


**Figure S6. Generation of KdmB* and *kdmB*Δ/*kdmB*^Cis^ mutants.** **(A)** Mutation and verification strategy of KdmB* in *Aspergillus nidulans*. Two conserved amino acids were mutated using primer harboring the desired mutations (indicated as a red dot). *In loco* insertion was checked via amplifying the 3’flank (ribo_fum_check-F//8211_downstream R). Primers are indicated in green. Correct mutation was checked via sequencing (data not shown). **(B)** Complementation and verification strategy of *kdmB*Δ*/kdmB*^Cis^ mutants in *A. nidulans*. Complementation *via* homologous recombination in the *kdmB*Δ strain with the *afpyrG* selection marker was verified by amplification of 5’ (dia_kdmB_F02//kdmB_comp_R2) and 3’ (dia_kdmB_F01//ribo_fum_Check_F) flanks for four independent transformants as well as inability to grow on riboflavin (data not shown). The *kdmB*∆ was used as a negative control. Diagnostic primers are indicated in green. M, marker (Gene ruler 1kb Thermo Fisher); -, no template control.

**Figure S7**


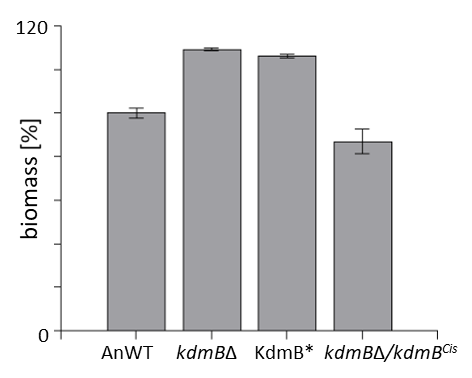


**Figure S7. Biomass production is significantly increased in *Aspergillus nidulans* *kdmB*Δ and KdmB*.** The *A. nidulans* wild-type strain (AnWT), *kdmB*∆, KdmB* and *kdmB*∆/*kdmB*^Cis^ were grown in liquid AMM for 48 h at 37 °C, 180 rpm in the dark. Subsequently, mycelia was harvested and lyophilized prior to biomass determiantion. Experiments were performed in technical triplicates. For comparison, the AnWT was arbitrarily set to 100 %. Mean values and standard deviations are shown.

**Figure S8**


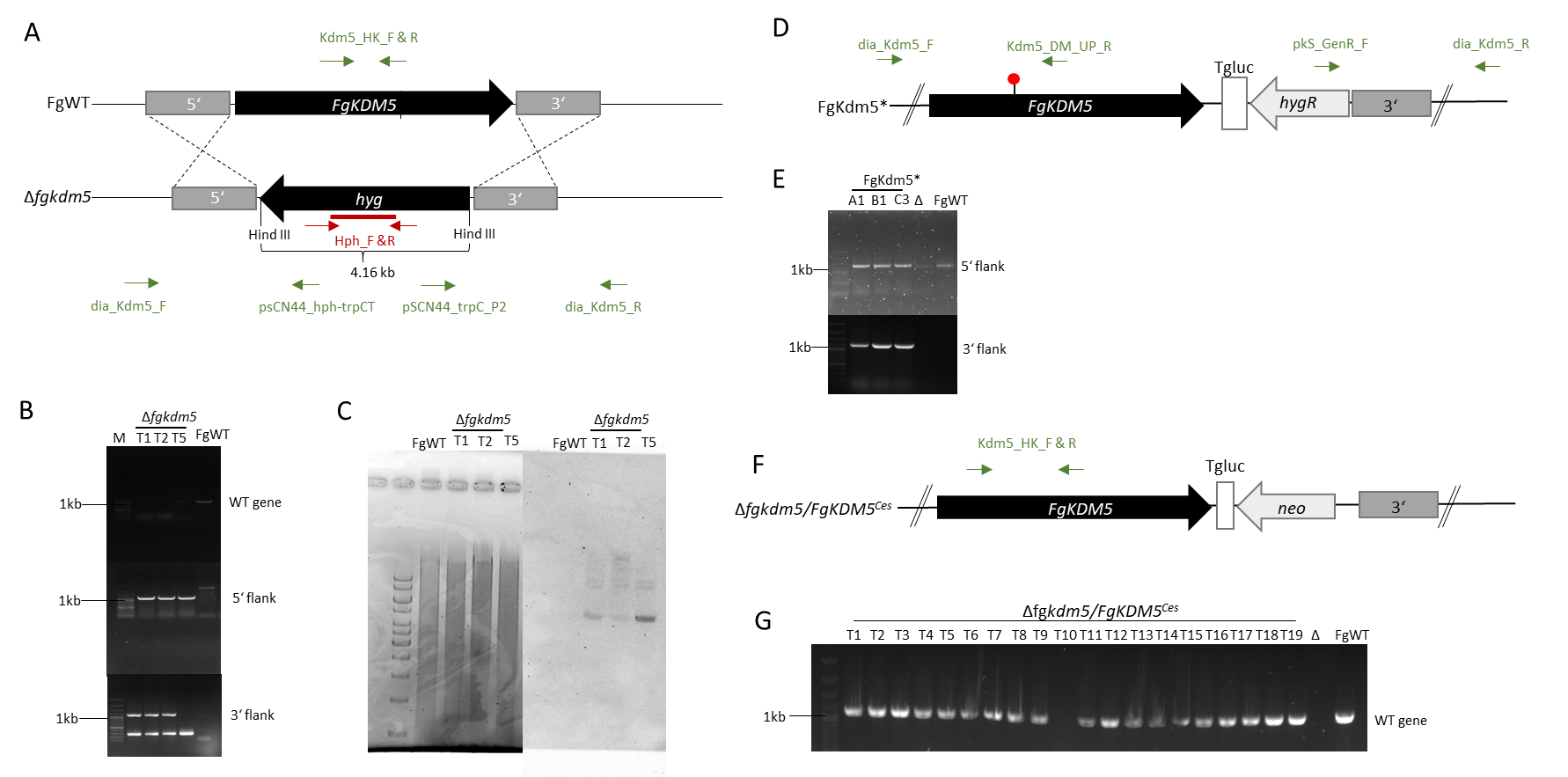


**Figure S8. Verification of *Fusarium graminearum* Δ*fgkdm5,* FgKdm5* and Δ*fgkdm5/FgKDM5^Ces^* mutants by diagnostic PCR and Southern blot.** **(A)** Deletion and verification strategy for Δ*fgkdm5* mutants in *F. graminearum*. Primers for diagnostic PCR are indicated in green. The probe for analyzing ectopic integration events by Southern blotting was amplified with the primer pair Hph-F//Hph-R. *Hin*dIII was used for genomic restriction digest. **(B)** Deletion *via* homologous recombination with the *hph* resistance cassette was verified by amplifying the 5’ (dia_Kdm5_F//psCN44_hph-trpCT) and 3’ (dia_Kdm5_R//psCN44_hph-trpC_P2) flanks as well as absence of the wild-type gene (Kdm5_HK_F//Kdm5_HK_R) for three independent transformants. The *F. graminearum* wild-type strain (FgWT) served as a control. **(C)**Genomic DNA of transformants and FgWT was digested with *Hind*III, while the hygromycin resistance cassette (hph) was used for probing. Detected signals match the expected 4.16 kb for the correct mutants, while no signal was detectable in FgWT. **(D)**Verification strategy of FgKdm5* mutants. Primers for diagnostic PCR are indicated in green. The desired mutation is indicated (red dot). **(E)**Integration of the mutated *FgKDM5* *via* homologous recombination with the hph resistance cassette was verified by amplification of 5’ (dia_Kdm5_F//Kdm5_DM_UP-R) and 3’ (dia_Kdm5_R//pkS_GenR_F) flanks for three independent FgKdm5* mutant strains. No 5’ and 3’ signal was detected for Δ*fgkdm5* and no 3‘ flank signal was detected for FgWT. **(F)**Verification strategy of Δ*fgkdm5/FgKDM5^Ces^* mutants by diagnostic PCR. **(G)** Integration of *FgKDM5* with the G418 (*neo* resistance cassette was verified by amplifying the wild-type gene’ (Kdm5_HK_F//Kdm5_HK-R) and inability to grow on hygromycin. Genomic DNA of Δ*fgkdm5* served as control. M, GeneRuler 1 kb DNA Ladder, Thermo Fisher Scientific.

**Figure S9**


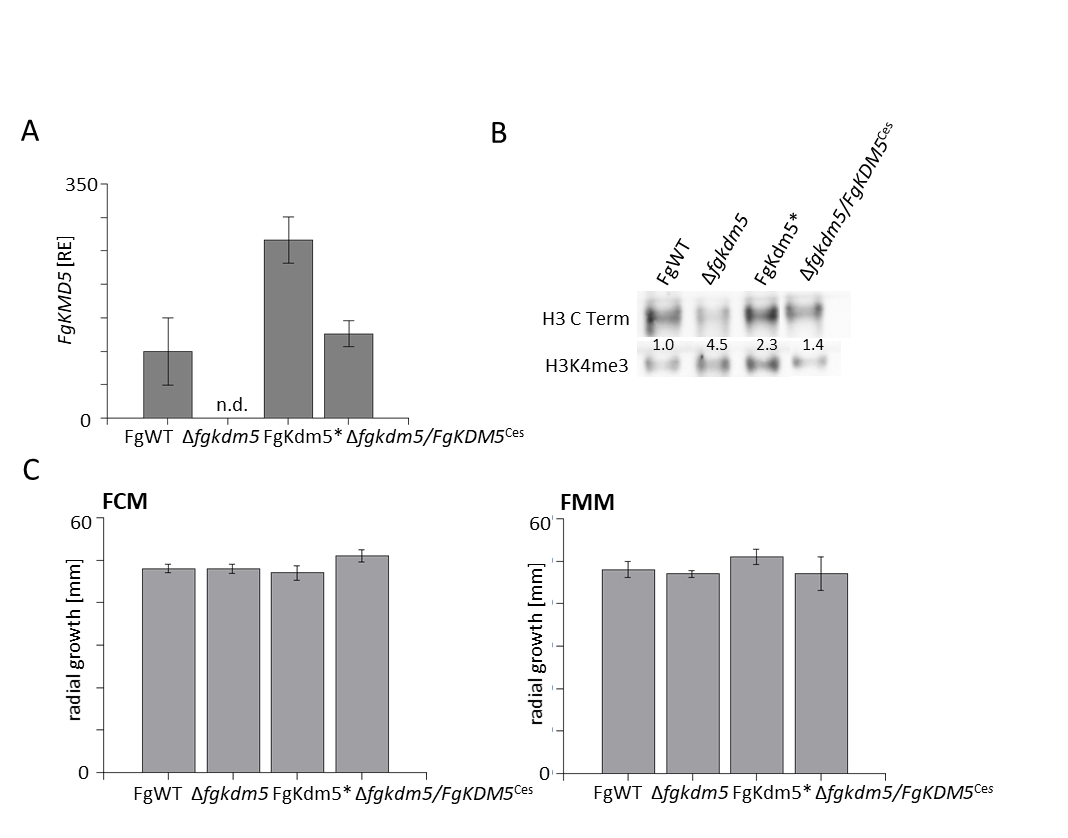


**Figure S9. Phenotypic characterization of *Fusarium graminearum* ∆*fgkdm5*, FgKdm5* and ∆*fgkdm5/FgKDM5*^Ces^ strains. (A)**The *F. graminearum* wild type (FgWT), ∆*fgkdm5*, FgKdm5* and ∆*fgkdm5/FgKDM5*^Ces^ strains were grown on Fusarium complete media (FCM) for 3 days at 20 °C in the dark. Total RNA was extracted from lyophilized mycelium and cDNA synthesis was generated prior to gene expression analysis. Experiments were performed in technical replications. Mean values and standard deviations are shown. RE, relative expression **(B)** Western analysis of indicated strains. The indicated strains were grown on FCM for 3 days prior to protein extraction. For the detection of H3K4me3 and H3 C-Term, 15 μg of the total protein extract was loaded onto the SDS gel. For relative quantification a densitometric analysis was performed. Signal intensities were normalized to the H3 C-Term control. **(C)**Radial growth of indicated strains was measured of the indicated strains after five days of growth on FCM and FMM plates at 20°C in the dark. Experiments were done in triplicates. Mean values and standard deviations are shown.

**Figure S10**

**
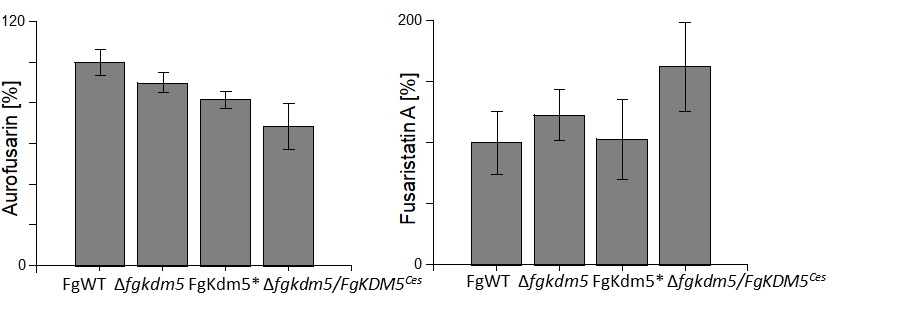
**

**Figure S10. FgKdm5 does not affect the biosynthesis of aurofusarin and fusaristatin A.** For SM analysis, the *F. graminearum* wild type (FgWT), Δ*fgkdm5*, FgKdm5* and Δ*fgkdm5/FgKDM5*^Ces^ strains were grown on PDA plates for two weeks at 25 °C in the dark. Subsequently, agar plugs were extracted and applied for HPLC-MS/MS analysis. Experiments were performed in triplicates with three independent mutants and secondary metabolite production of the respective wild-type strains was arbitrarily set to 100 %. Mean values and standard deviations are given.

**Figure S11**

**
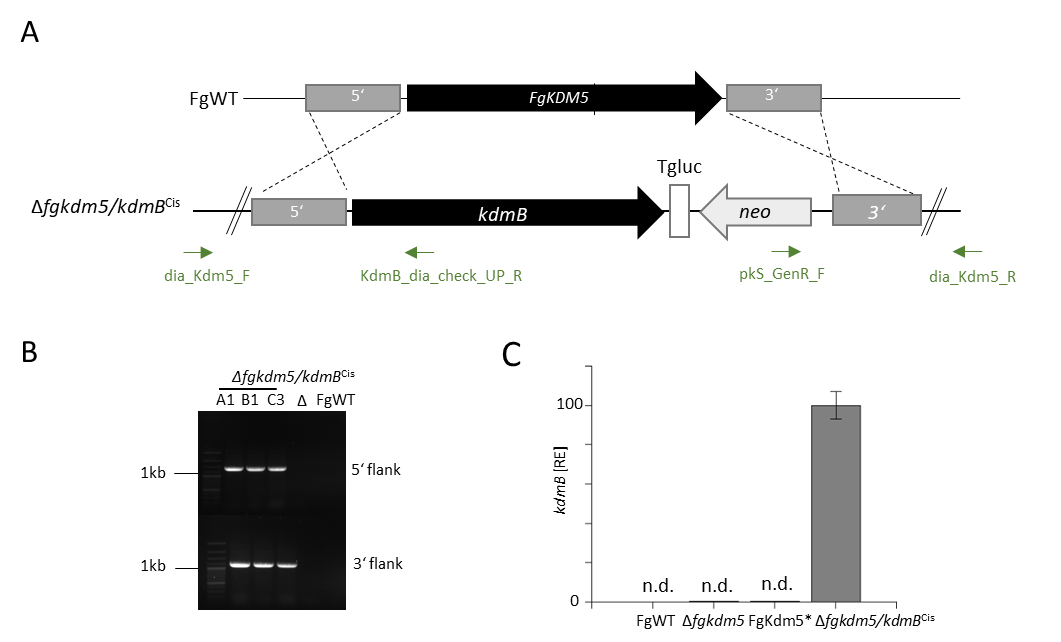
**

**Figure S11. Verification of Δ*fgkdm5/kdmB*^Cis^ mutants by diagnostic PCR and RT-qPCR.** **(A)**Verification strategy of Δ*fgkdm5/kdmB^Cil^* mutants. Primers used for verification are indicated in green. **(B)** Integration of the *A. nidulans* *kdmB* gene *via* homologous recombination with the hph resistance cassette was verified with the amplification of 5’ (dia_Kdm5_F/KdmB_dia_check_UP_R) and 3’ (dia_Kdm5_R/ pkS_GenR_F) flanks for three independent Δ*fgkdm5/kdmB*^Cis^ mutants. Both Δ*fgkdm5* and FgWT served as negative control. **(C)** For additional verification, the *kdmB* gene expression was checked in Δ*fgkdm5/kdmB*^Cis^ mutants. Prior to RNA extraction, indicated strains were grown on FCM at 20 °C for three days in the dark. Experiments were done in technical replicates. Mean values and standard deviations are shown. RE, relative expression.

**Figure S12**

**
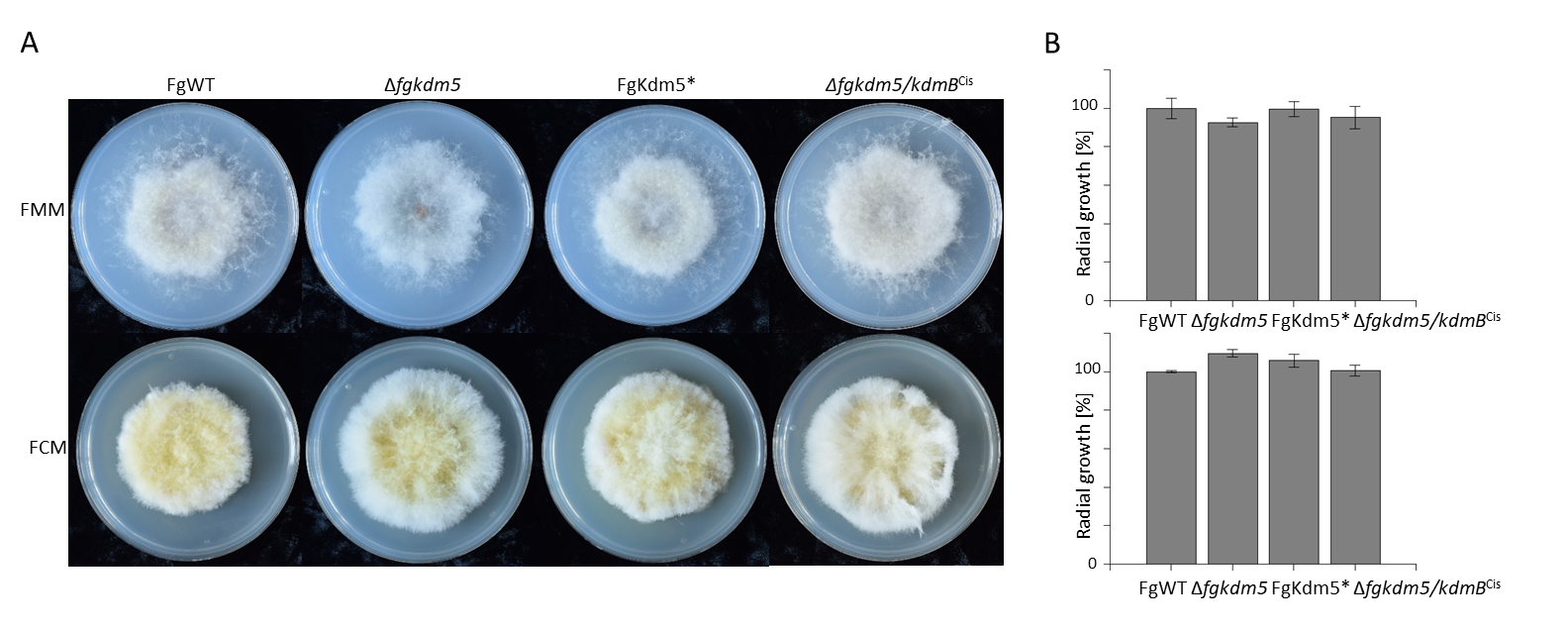
Figure S12. Cross-complementation strains (∆*fgkdm5/kdmB*^Cis^) did not show an altered growth phenotype compared to the *F. graminearum* wild-type strain (FgWT).** For radial growth assays, FgWT, ∆*fgkdm5*, FgKdm5* and ∆*fgkdm5/kdmB*^Cis^ were grown on Fusarium minimal (FMM) and complete medium (FCM) for five days at 20 °C in the dark. Pictures were taken five days post inoculation (A) and radial growth was measured (B).

**Figure S13**


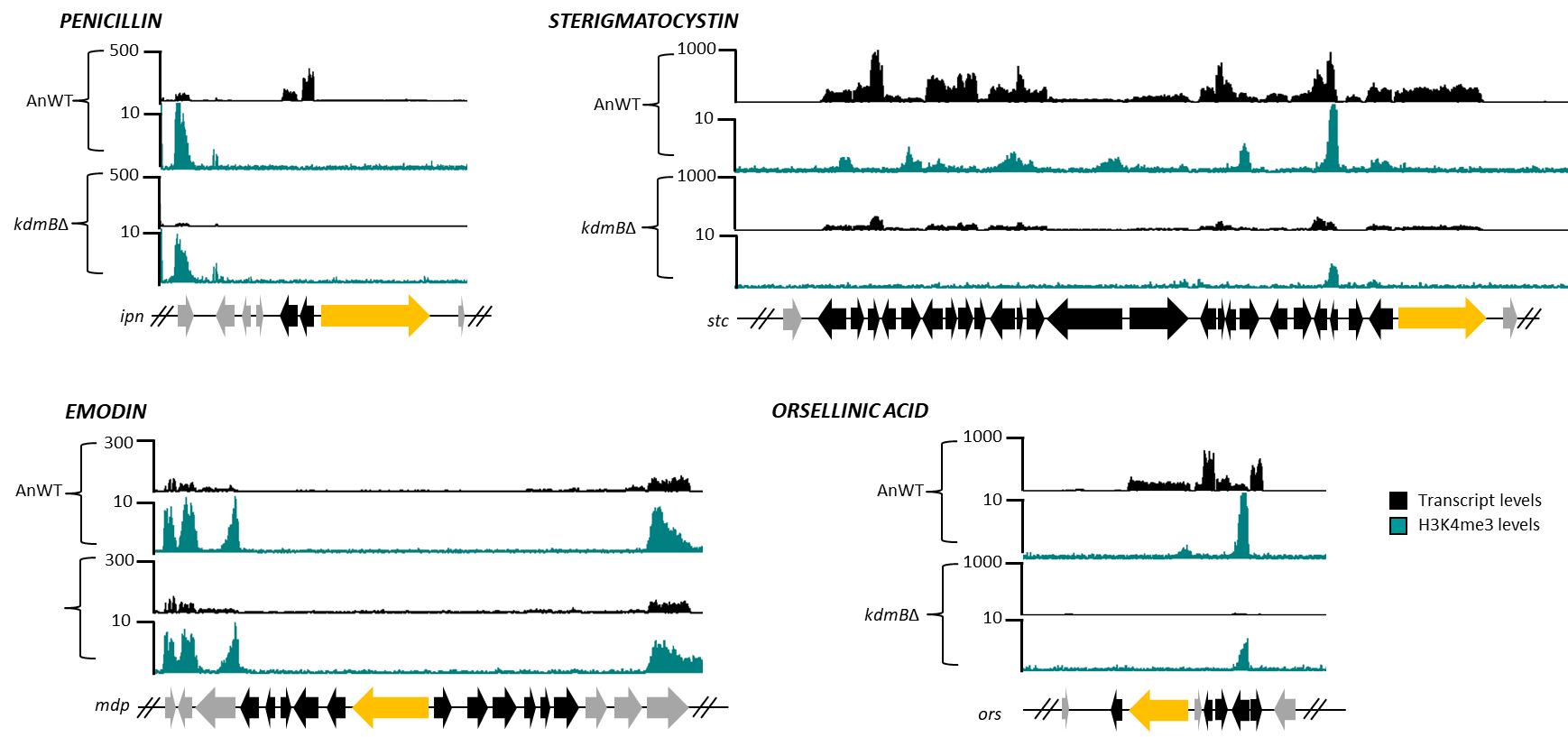


**Figure S13. Secondary metabolite gene clusters (SMGCs) are largely devoid of H3K4me3 in *Aspergillus nidulans*.** Transcript (black) and H3K4me3 (green) levels are shown for analyzed SMGCs of the *A. nidulans* wild-type strain (AnWT) and *kdmB*Δ under SM-inducing conditions (48 hrs in AMM with 10 mM sodium nitrate). Black arrows indicate genes within the cluster; key enzyme-encoding genes in this study are indicated in yellow. Genes indicated in grey do not belong to the SMGC (modified from Gacek-Matthews et al. 2016).

**Table S1 Primers used in this study**

| ***Primer ID*** | ***Primer sequence*** |  |
| --- | --- | --- |
| ***Primers used for* A. nidulans *plasmid generation*** | | |
| UP_F1 | gtaacgccagggttttcccagtcacgacgtcccacccctctagaggctg |  |
| UP_R1 | gcagagtaagcgtaatgatcctggttgccccagcaaaaggttgagaagc |  |
| Middle_F2 | ttggaggccgttcaggagtcc |  |
| Middle_R2 | ggactcctgaacggcctccaa |  |
| Ribo_F3 | atccgaaccctcatacaccgaaatgagtgtgacgagcatacaccg |  |
| Ribo_R3 | gtaggtgtggcttcgtgattggaggccgttcaggagtccgg |  |
| Down_F4 | cggtgtatgctcgtcacactcatttcggtgtatgagggttcgg |  |
| Down_R4 | gcggataacaatttcacacaggaaacagcggcttcaaactactcgagaa |  |
| kdmB_comp_F1 | gtaacgccagggttttcccagtcacgacgACGCGTTTGCTTCAGACTGTGTTGCTCATGTAGTCG |  |
| kdmB_comp_R1 | TTCTTGGGCATAGGCGAATG |  |
| kdmB_comp_F2 | CATTCGCCTATGCCCAAGAATCCCACCCCTCTAGAGGCTGAGA |  |
| kdmB_comp_R2 | ATATTGCATCTACAGCTGCG |  |
| kdmB_comp_F3 | CGCAGCTGTAGATGCAATAT |  |
| kdmB_comp_R3 | CAATCACGAAGCCACACCTAC |  |
| kdmB_comp_F4 | GTAGGTGTGGCTTCGTGATTGGCCTCAAACAATGCTCTTCAC |  |
| kdmB_comp_R4 | ATTCTGTCTGAGAGGAGGCAC |  |
| kdmB_comp_F5 | GTGCCTCCTCTCAGACAGAATTTCGGTGTATGAGGGTTCGGATTG |  |
| kdmB_comp_R5 | gcggataacaatttcacacaggaaacagcACGCGTGAAGTGAGGATGTTTGAGAAGCCTGTGG |  |
| AnCclA_del_01F | gttttcccagtcacgacgTCCTCATTGAACTTGCCTTCGTCGTCCG |  |
| AnCclA_del_01R | TGAAGAGCATTGTTTGAGGCAATGCGAATCCTTTGCGGATAGG |  |
| AnCclA_del_02F | GCCTCCTCTCAGACAGAATATGGGGTTGTACTTTCAGCTGCCTGCGTGG |  |
| AnCclA_del_02R | caatttcacacaggaaacagcACATCTCTGAACTCGCCCCTTCAGTGC |  |
| SppA_del_F1 | cagggttttcccagtcacgacgCCCCCCGAGGCTTTCTCCACCAGCTC |  |
| SppA_del_R1 | TGAAGAGCATTGTTTGAGGCTTCGAGCTGCGGGCCGGGGACCTGCGAT |  |
| SppA_del_F2 | GCCTCCTCTCAGACAGAATGCTGCATGCCGTCTTGAGTTCCGTGGACTCG |  |
| SppA_del_R2 | caatttcacacaggaaacagcAAGCCCCTCCAATTTCCCGCAGCGAAGC |  |
| pyrG_fum_F | GCCTCAAACAATGCTCTTCA |  |
| pyrG_fum_R | ATTCTGTCTGAGAGGAGGCACTGA |  |
| ***Primers used for* F. graminearum *plasmid generation*** | | |
| Kdm5_5F | CGCCAGGGTTTTCCCAGTCACGACGCTCAGCGCTGCAACAACTACTAGC |  |
| Kdm5_5R | ATCCACTTAACGTTACTGAAATCTCCAAGGAAACGTATTGGGGTATCG |  |
| Kdm5_3F | TCCTTCAATATCATCTTCTGTCTCCGACGAGGGTAATATTGGTGCAGGG |  |
| Kdm5_3R | TAACAATTTCACACAGGAAACAGCCGTACCATTCACAGGCAAGGTAAGC |  |
| Hph-F | GTCGGAGACAGAAGATGATATTGAAGGAGC |  |
| Hph-R | gttggagatttcagtaacgttaagtggat |  |
| FgKDM5-Dmil_F | cgccagggttttcccagtcacgacgCTTCACCAATACATCCAAGCTGC |  |
| FgKDM5_H641G-E643Q_F | GCGTAATGATCCTGGTTACCCCAACAGAATG |  |
| FgKDM5_H641G-E643Q_R | CATTCTGTTGGGGTAACCAGGATCATTACGC |  |
| Kdm5_Tgluc_ORF | CATACATCTTATCTACATACGTCACTCTCCATCAAGCAAAGC |  |
| Tgluc-nat1R | ATCTTGTTGGGGGGAAGGGGT |  |
| Tgluc_F2 | CGTATGTAGATAAGATGTATG |  |
| FgKdm5_F03 | GCCATCACTCAAGTCACTGCGTG |  |
| FgKdm5_R03 | CACGCAGTGACTTGAGTGATGGC |  |
| Geni-Tgluc_R | ACCCCTTCCCCCCAACAAGATATCATCATGCAACATGCATGTACTG |  |
| GeniF | GTCGGAGACAGAAGATGATATTGAAGGAGCCAACAAAACACAGTTCCGACCAC |  |
| Kdm5_R02 | CGGGTGAGTCGCCTCGAAGACTGACAGGC |  |
| Kdm5_F02 | GCCTGTCAGTCTTCGAGGCGACTCACCCG |  |
| Kdm5_R03 | CACGCAGTGACTTGAGTGATGGC |  |
| Kdm5_F03 | GCCATCACTCAAGTCACTGCGTG |  |
| AnKdmB1F | atggtggctccggctgcaatgg |  |
| AnKdmB1R | cccatagcaagtcttggtagctc |  |
| AnKdmB2F | ggagctaccaagacttggtatgg |  |
| KdmB_CrossCil_R | CATACATCTTATCTACATACGTTAAGCGGCCGCAGTGGCTTCGACTTCC |  |
| pFgKdm5UP-AnKDMB_R | CCATTGCAGCCGGAGCCACCATGGCGAACGCGGATGATGATGGATGG |  |
| genR_split_F | GGGAAGGGACTGGCTGCTATTG |  |
| genR_split_R | GCAATATCACGGGTAGCCAACG |  |
| Splitmarker_hph_F | cgttgcaagacctgcctgaa |  |
| Splitmarker_hph_R | ggatgcctccgctcgaagta |  |
| ***Primers for diagnostic PCR*** | | |
| Ribo_fum_check_F | gtgctggaagtacgccttcctg |  |
| 8211_downst_R | GATTCGTCATCGCCTTCAG |  |
| Dia_kdmB_F02 | AGTCAATGGTCAACCTGAGC |  |
| Dia_kdmB_R02 | CAGTGCGAAGGACAAAGCAGC |  |
| Dia_pyrG_R01 | ATTCTGTCTGAGAGGAGGCACTGA |  |
| Dia_kdmB_F01 | CGATTCGTCATCGCCTTCAGC |  |
| dia_dCclA_UP_F | CTGACCACATACCACTTCCTC |  |
| dia_dCclA_DownR | CTTGCCTCAGCTCACCTCTC |  |
| dia_dSppA_UP_F | CTCAATCTGGCGCATTTGGC |  |
| dia_dSppA_DownR | CAATGCCATCTTTGCCCCCGC |  |
| dia_pyrG_fum_5' | GCCATGAAGCGCCAATTGCTG |  |
| dia_pyrG_fum_3' | GACCTGTGCAGAAGAGATAACAG |  |
| dia_WTCclA_F | GTCGTTGATAAACTTACGAACAAG |  |
| dia_WTCclA_R | GCTCACCGAGCCCGAGGTTG |  |
| dia_WTSppA_F | CATGGAGAAAGGCAAGCATATGC |  |
| dia_WTSppA_R | GACCGGTCTGACGACGACGCCCG |  |
| Dia_kdm5_F | CAGGGAAAGAGCTCACATTCTC |  |
| Dia_kdm5_R | GAAAGCCTCTGCTAACAAG |  |
| pSCN44-trpC_P2 | gtgatccgcctggacgactaaacc |  |
| pCSN44-hph-trpC-T | ggaatagagtagatgccgaccgg |  |
| Kdm5-DM-UP_R | CCGTTTGAGTTGACTTTCGGC |  |
| pkS-Gen-gpd_P | ggtgatgagcaggtggtgagagg |  |
| KdmB_dia_check_UP-R | CGGAAAGTAGGTGCCTCTTGC |  |
| Kdm5_HK_F | GTTGAACCCGGCATGGTAAG |  |
| Kdm5_HK_R | CGTCAATTCCCATTGTCGCATAC |  |
| ***Primers used for Southern probe amplification*** | | |
| AnCclAF | CGGGACTCAAAATCGAAGGC |  |
| AnCclA_UP_R | CCTTTGCGGATAGGTATTTCACC |  |
| AnSppA_down_F | GAGTTCCGTGGACTCGAATTCAG |  |
| AnSppA_down_R | GCCCCTCCAATTTCCCGCAGCG |  |
| Hph_F | GTCGGAGACAGAAGATGATATTGAAGGAGC |  |
| Hph_R | gttggagatttcagtaacgttaagtggat |  |
| ***Primers used for sequencing*** | | |
| **pFgKdm5*** | | |
| prs426_seq | cgctattacgccagctggc |  |
| Kdm5_up_seq01 | GAAGTCGATGCACGCCGCG |  |
| Kdm5_seq_02 | CGTTGATAAGAAACCGC |  |
| Fgkdm5_Mut_1F | GATGGCCAGTTTGGTTTCG |  |
| Kdm5_seq_F03 | GCCGGATCTCCTCTTCCAG |  |
| Kdm5_seq_F04 | GATCTGACTACACGATGTCGCG |  |
| Kdm5_seq_F05 | GAACTCCACAAGTGGTCTCCTG |  |
| Kdm5_seq_F06 | GCATCCAAACGGATTCTG |  |
| T-gluc-seq | cgagccagactcctgaacggcct |  |
| Gen-seq1 | GAGCCTGAATGTTGAGTGG |  |
| Gen-seq3 | CAGCCGATTGTCTGTTGTGC |  |
| prs426.REV | gttgtgtggaattgtgagcgg |  |
| **pΔ*fgkdm5/kdmB^Cil^*** | | |
| prs426_seq | cgctattacgccagctggc |  |
| Kdm5_up_seq01 | GTCCACGAACCAGTGACCTTG |  |
| kdmB(anid)cdsbein.For | TATCGACTGTCGAAAGACGAG |  |
| AN8211_orfF | AATCCCACCCCTCTAGAGGCTG |  |
| Seq_F9 | GAGTTCGATTGGCATTGC |  |
| KdmB(anid)midFor | CTGAAGCATTCGAGGAAGCAATGCGACAAGCGGTCCCTGAACT |  |
| KdmB(anid)midSFor | CAGCTGGAAGAACGTGAC |  |
| KdmB(anid)mid2For | GTGGACAGTCGAATGACAG |  |
| kdmB_comp_F3 | CGCAGCTGTAGATGCAATAT |  |
| kdmB(anid)endFor | GCTCGAATGGGTATCCAAAG |  |
| kdmB(anid)endRev | GGTATCGATAGACTTGCGC |  |
| tglucF2 | CGTATGTAGATAAGATGTATG |  |
| geniR_splitR | GCAATATCACGGGTAGCCAACG |  |
| genseq3 | CAGCCGATTGTCTGTTGTGC |  |
| prs426.REV | gttgtgtggaattgtgagcgg |  |
| **pΔ*kdmB/kdmB^Cil^*** | | |
| seqR4 | cgaagccacacctacaaagt |  |
| seqR5 | atctgcggagacatgaggtag |  |
| seqR6 | tgtcacagtgtcatggtcag |  |
| seqR7 | ctggcagtccttgagagtca |  |
| seqR8 | TGAGTCAAGAAGATGTAGGC |  |
| seqR9 | TATGACGGAACAGAGACTCC |  |
| seqR10 | CGATGAACGAGGGTGATT |  |
| seqF9 | GAGTTCGATTGGCATTGC |  |
| kdmB_comp-R1 | TTCTTGGGCATAGGCGAATG |  |
| **pΔ*fgkdm5/kdm5^Cel^*** | | |
| Kdm5_up_seq_01 | GAAGTCGATGCACGCCGCG |  |
| Kdm5_seq_02 | CGTTGATAAGAAACCGC |  |
| Kdm5_F02 | GCCTGTCAGTCTTCGAGGCGACTCACCCG |  |
| Kdm5_Hk_dia_R | CGTCAATTCCCATTGTCGCATAC |  |
| kdm5_seq_F03 | GCCGGATCTCCTCTTCCAG |  |
| kdm5_seq_F04 | GATCTGACTACACGATGTCGCG |  |
| kdm5_seq_F05 | GAACTCCACAAGTGGTCTCCTG |  |
| Fgccl1_cc_seq1 | tcaggctgcgcaactgttgg |  |
| Tgluc_F2 | CGTATGTAGATAAGATGTATG |  |
| Geni_split_F | GGGAAGGGACTGGCTGCTATTG |  |
| Geni_split_R | GCAATATCACGGGTAGCCAACG |  |
| ptrpC | acagaagatgaTattgaaggagc |  |
| prs426.REV | gttgtgtggaattgtgagcgg |  |
| An_aflR_seq5 | CATACATCTTATCTACATACG |  |
| ***Primers for RT-qPCR*** | | |
| aflR_F | AGCCCAGCTGGTGCTGAGCGAGCTATAC |  |
| aflR_R | CCAGGGTGGTCGACGACAAGGGGGT |  |
| ipna_F | GGAGACGACCAAGCAGCCAAA |  |
| ipna_R | TTTTCCCGGGGATGGACAGG |  |
| orsA_F | GCACTGCTGTTCTATTGCC |  |
| orsA_R | CAGCTTCCAGCCATGATTAAG |  |
| mdpG_F | GCACCGGCGTCAGTTACTCCAG |  |
| mdpG_R | CCAAAGCCCAGCGCAGC |  |
| q_ActA_fwd | atgtcgacgtccgtaaggatctg |  |
| q_ActA_rev | ttccttctgcatacggtcggag |  |
| q_tub_F | GATGGCTGCCTCTGACTTCCG |  |
| q_tub-R | GCGCATCTGGTCCTCAACCTC |  |
| q_kdmB_F | CTTACCAACCAGGATGCCGACCC |  |
| q_kdmB_R | CGACTTCCGGCTCAGGTTGACC |  |
| q_FgKdm5_3’_F | GGCCCAGCTCGGTTCTTGAAAGG |  |
| q_FgKdm5_3’_R | CCGCACCAGGGACAACTCCATTAACC |  |
| GAPDH_qPCR_fwd | CGTCAACGGCAAGACCATCAAGTT |  |
| GAPDH_qPCR_rev | CCCTTCTCGAGGCGAACAGTCAA |  |
| qPCR_actin_F | TGGTGGTACCACCATGTACCCCG |  |
| qPCR_actin_R | GGGAAGCGAGAATGGAACCACCG |  |
| cDNA_ß-TUB_F | TGCGCTATTCCCCCTCGTGG |  |
| cDNA_ß-TUB_R | TGGACTCGGCCTCAGTGAACTCC |  |

Table S2 Strain generation strategies.

| KdmB* | *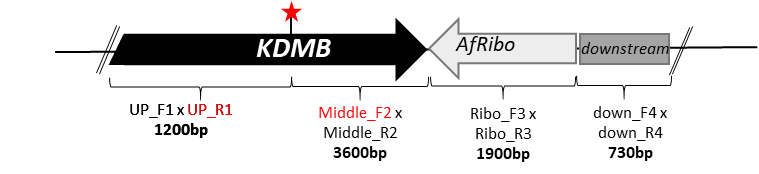* |
| --- | --- |
| *kdmB*Δ/*kdmB*^Cis^ | *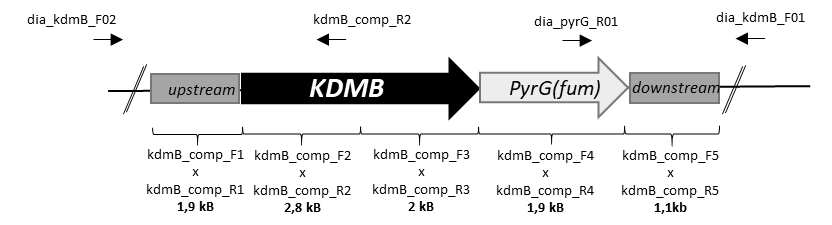* |
| *cclA*Δ | *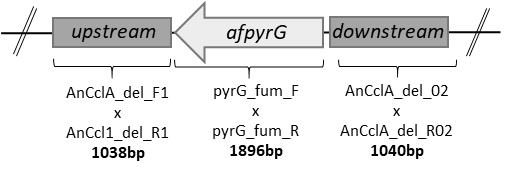* |
| *sppA*Δ | *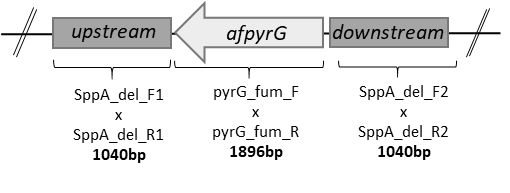* |
| Δ*fgkdm5* | *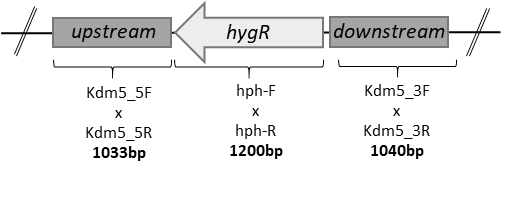* |
| FgKdm5* | *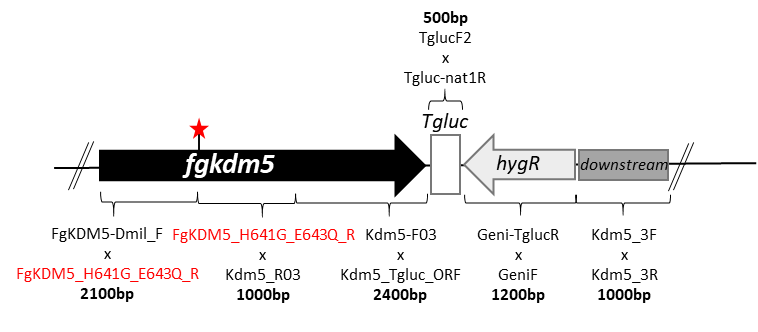* |
| Δ*fgkdm5/kdmB^Cis^* | *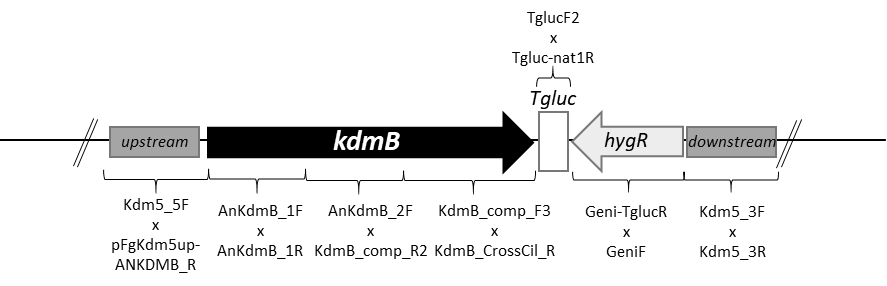* |
| *Δfgkdm5/KDM5^Ces^* | *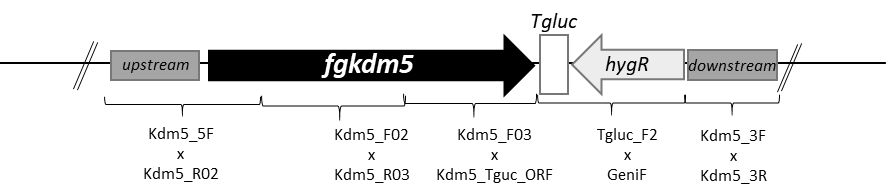* |
